# Effects of social and nonsocial reward on executive function in preschoolers

**DOI:** 10.1002/brb3.1763

**Published:** 2020-07-30

**Authors:** Kanda Lertladaluck, Nuanchan Chutabhakdikul, Nicolas Chevalier, Yusuke Moriguchi

**Affiliations:** ^1^ Research Center for Neuroscience, Institute of Molecular Biosciences Mahidol University Nakhon Pathom Thailand; ^2^ Department of Psychology The University of Edinburgh Edinburgh UK; ^3^ Graduate School of Education Kyoto University Kyoto Japan

**Keywords:** children, executive function, near‐infrared spectroscopy, prefrontal cortex, reward

## Abstract

**Introduction:**

Executive function, a set of higher order cognitive skills underlying goal‐directed behaviors, develops rapidly during preschool years. Reward increases executive function engagement in adolescents and adults. However, there is still a scarcity of data on how reward affects executive function in young children. The present study examines whether different incentive types contribute differently to executive function performance and neural activity in children.

**Methods:**

Twenty‐five preschoolers of 5–6 years old were provided an incentive Go/No‐go task, comparing social, nonsocial, and nonreward conditions. Activations in the prefrontal regions during the tasks were measured using functional near‐infrared spectroscopy.

**Results:**

The results revealed that social reward enhanced right prefrontal activations in young children. In contrast to adult literature, younger children did not show any significant differences in executive function performance across conditions.

**Conclusion:**

This study expands our understanding of motivation and EF engagement in preschoolers. Specifically, social reward enhanced prefrontal activations in young children. The implications and recommendations for future research are discussed.

## Graphical abstract

We examined the effect of social and nonsocial reward on EF engagement and neural activity in 5‐ to 6‐year‐old children. The results revealed that social reward enhanced prefrontal activations in the right inferior frontal gyrus although children did not show any significant differences in executive function performance across conditions.

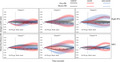

## 1 INTRODUCTION

Executive function (EF) broadly refers to a set of higher order cognitive control processes that are involved in goal‐directed behaviors and serve cognitive functions, such as working memory, inhibitory control, attention shifting, and planning (Diamond, 2013; Garon, Bryson, & Smith, 2008). EF is related to various aspects of child functioning, such as school readiness and success (Blair & Razza, 2007), theory of mind (Hughes & Ensor, 2005), and social–emotional competence (Moriguchi, Okanda, & Itakura, 2008; Riggs, Jahromi, Razza, Dillworth‐Bart, & Mueller, 2006). The preschool period (3–6 years of age) is the stage of important development in EF task performance (Diamond, 2013). Previous neuroimaging studies have highlighted the key role of the prefrontal cortex (PFC), more specifically the lateral prefrontal regions, during EF tasks, such as working memory, inhibitory control, and cognitive flexibility in preschool children (e.g., Moriguchi & Hiraki, 2009, 2013; Tsujimoto, Yamamoto, Kawaguchi, Koizumi, & Sawaguchi, 2004). Moreover, the connections of the PFC within itself and with other cortical and subcortical brain structures form a system that subserves EF (Heyder, Suchan, & Daum, 2004).

Previously, Zelazo and colleagues proposed a framework of cool and hot distinction in EF (Zelazo & Carlson, 2012; Zelazo & Müller, 2002), in which cool EF is engaged in neutral, and nonaffective situations. On the other hand, hot EF processes are elicited under affective conditions, such as a delay of gratification (Moriguchi, Shinohara, & Yanaoka, 2018). Thus, the key differences between hot and cool aspects are whether EF is invoked with or without a motivationally or emotionally salient context. For example, in the delay of gratification task, children have to forgo a small immediate reward to obtain a larger delayed reward. In this task, children engage inhibitory control in the face of reward (motivationally salient context). However, there is still a scarcity of data on how EF performance and its neural correlates differ with and without motivational context in young children. The present study examined the issue.

In general, motivation can be defined as the reason that drives individuals to conduct certain tasks. This is also claimed as a process that initiates, guides, and maintains goal‐directed behaviors. In terms of developmental psychology, fostering intrinsic motivation in the early years may have long‐lasting and self‐sustaining effects. Nevertheless, the appropriate use of external rewards, which is the external stimuli driving an appetite to alter behavior, is also necessary for children with little intrinsic motivation (Carlton & Winsler, 1998; Howard‐Jones & Jay, 2016). These external rewards include both tangible rewards (e.g., toys, stickers, money) and intangible rewards (e.g., verbal praise, smiles). Recently, there has been much interest in the beneficial effect of motivation on EF performance. Several studies in preadolescence through adulthood have shown that high reward signals and motivational state could improve EF performance. For example, stimulus salience and reward expectations can enhance visual working memory capacity, especially during information encoding (Klink, Jeurissen, Theeuwes, Denys, & Roelfsema, 2017). Rewards may similarly affect response inhibition, as suggested by improved response inhibition accuracy in preadolescents aged 8–12 years when completing the Go/No‐go task with reward contingencies (Kohls, Peltzer, Herpertz‐Dahlmann, & Konrad, 2009).

Neuroimaging evidence shows that motivation and reward‐related cognition are supported by a reward system that includes the ventral tegmental area, substantia nigra, amygdala, orbitofrontal cortex, medial PFC, insular cortex, anterior cingulate cortex, and ventral striatum (Haber & Knutson, 2010). These brain areas play different roles in the reward processing. For example, neural activation in the ventral striatum supports reward anticipation, while neural activation in the ventromedial PFC supports processing of reward outcomes (Knutson, Fong, Adams, Varner, & Hommer, 2001). There are multiple ways in which reward processing may influence PFC activity and thus EF engagement, including (a) tonic dopamine release in the mesolimbic and mesocortical pathway (Beierholm et al., 2013), (b) strengthening direct connectivity between the ventromedial and the dorsolateral PFC (Barbas & Pandya, 1989; Kouneiher, Charron, & Koechlin, 2009), (c) ventromedial to dorsolateral direction of information flow through the frontostriatal–nigral circuitry (Haber & Knutson, 2010), and (d) the engagement of certain brain areas such as anterior cingulate cortex (Shenhav, Botvinick, & Cohen, 2013).

Recent studies on monetary and social rewards revealed that different incentive types have diverse motivational effects (Demurie, Roeyers, Baeyens, & Sonuga‐Barke, 2012; Kohls, et al., 2009; Lin, Adolphs, & Rangel, 2012; Rademacher et al., 2010; Spreckelmeyer et al., 2009). Most of these studies have focused on adult participants. At the behavioral level, the results indicated greater performance with monetary reward than social reward (e.g., happy face). One possible explanation for less effectiveness of social incentives is that social reward may act as immediate rewards with short‐term effects (i.e., they cannot be accumulated over time and subsequently exchanged for a prize). By contrast, financial rewards may have long‐term effects, as the rewards can be stocked up and subsequently exchanged for other items. Hence, financial rewards may be more desirable than social ones (Estle, Green, Myerson, & Holt, 2007). On the other hand, developmental studies have revealed that the incentive value of social reward, more specifically the tangible and quantitative social reward, is not less effective than monetary reward in children (Demurie et al., 2012; Wang, Liu, & Shi, 2017). Using face and nonface stimuli, children aged 6–8 years have been found to be more sensitive to social reward using face stimuli than the latter (Stavropoulos & Carver, 2014). Furthermore, the social stimuli have a heightened reward value at adolescence (Foulkes & Blakemore, 2016). Thus, it is possible that there are differences in reward processing among children, adolescents, and adults (Casey, Jones, & Hare, 2008).

Previous neuroimaging studies suggested that both social and nonsocial reward may share common neural substrates, such as ventromedial PFC and striatum (Izuma, Saito, & Sadato, 2008; Lin et al., 2012). Nevertheless, partially distinct neural activation patterns were observed for monetary and social reward (Flores, Munte, & Donamayor, 2015; Spreckelmeyer et al., 2009). More specifically, during reward consumption, the thalamus was more strongly activated by the presentation of monetary rewards while the amygdala was more strongly activated by the presentation of social feedback (Rademacher et al., 2010).

There has been a lot of interest in the beneficial effects of reward and, more broadly, motivation on EF performance in children, adolescents, and adults, but in young children this topic has mostly been examined at the behavioral level. It has been reported that preschoolers were responsive to motivational and emotional stimuli (Zelazo, Qu, & Kesek, 2010). For example, children aged 4–5 years who were informed about the reward they would receive performed better on the Day/Night Stroop task than children who were not informed about the reward (Qu, Finestone, Qin, & Reena, 2013). Moreover, the 3.5‐ to 4.5‐year‐old children were more accurate but had slower reaction time on the postswitch during dimensional change card sort (DCCS) when trial‐by‐trial, reward‐related feedback was provided (Tarullo, Nayak, St John, & Doan, 2018). Besides, using facial expressions as stimuli in the DCCS task instead of neutral objects can improve children cognitive flexibility (Qu & Zelazo, 2007). By contrast, in one behavioral study using a conflict EF task with either a cool (neutral) or hot (external reward) focus in children aged 2.5–4.5 years, no motivational valence differences were found on response accuracy (Beck, Schaefer, Pang, & Carlson, 2011). Thus, the effects of reward on EF performance in young children remain unclear. Although the abovementioned research has compared hot and cool EF by using the same tasks, most studies have typically assessed both hot and cool EF using different tasks (Hongwanishkul, Happaney, Lee, & Zelazo, 2005). Besides, it is unknown whether different incentive types underlying the hot aspects contribute differently to EF performance in preschool children, as is the case in adults. Given the paucity of behavioral findings, it is not surprising that no studies have examined the neural mechanisms underlying reward effects in preschool children.

The present study directly addressed the effect of social and nonsocial reward on EF in young children from age 5 to 6 years. To this end, we employed an incentive Go/No‐go task comparing social, nonsocial, and nonreward conditions in typically developing preschoolers. The task was conducted during near‐infrared spectroscopy (NIRS) measurement. Although NIRS may not be appropriate to measure deep brain regions associated with reward processing, previous studies using NIRS have shown that infants and adults also activate the medial frontal cortex (MFC) following exposure to rewarding stimuli, which likely reflects the reward system (Kida & Shinohara, 2013; Kringelbach, 2005; Minagawa‐Kawai et al., 2009). In a typical Go/No‐go task, activation in the right inferior frontal gyrus (IFG) supports EF engagement (Aron, Robbins, & Poldrack, 2014; Monden et al., 2015). Therefore, we measured activations in the lateral PFC and MFC to probe EF engagement and reward processing, respectively.

In the social reward condition, parental faces were used as feedback. Most facial expressions used in previous studies belonged to a stranger's face, obtained from a database (e.g., the NimStim set), which showed only little reward effects. However, brain responses to the mother's face differ from those to a stranger's face in infants and young children (Carver et al., 2003; Minagawa‐Kawai et al., 2009). An event‐related potentials (ERP) study in 4–6‐year‐old children indicated that the mid‐latency frontocentral negativity was larger for angry mothers’ faces than strangers’ emotional expressions (Todd, Lewis, Meusel, & Zelazo, 2008). Furthermore, the NIRS study shows that the MFC, which is part of the reward network, could be activated when infants viewed their own mother's smile (Minagawa‐Kawai et al., 2009). It is possible that for young children, parental faces can be more effective positive reinforcers than a stranger's face. In the nonsocial reward condition, although money is used as a reinforcer in many adult studies, the concept of money might not be fully established by preschool age (Berti & Bombi, 1981; Grunberg & Anthony, 1980). In this study, we used stickers, one of the most widely used positive tangible reinforcers, as feedback, which is similar to other previous hot and cool EF studies in young children (Tarullo et al., 2018).

In summary, based on aforementioned studies of reward and EF in children, we hypothesized that rewards would increase motivation in preschool children. This should be evidenced by greater MFC activation in the two reward conditions than in the control condition. In turn, this greater motivation may promote greater EF engagement, as reflected by both greater behavioral performance and greater lateral PFC activity. Moreover, we expected both social reward (smile from parents) and nonsocial reward (sticker) would enhance both EF performance and brain activity in preschoolers, relative to no reward. Also, reward sensitivity measured by the parent‐reported Behavioral Inhibition System/Behavioral Activation System (BIS/BAS) scale has been found to affect cognitive control performance in the monetary reward condition (Kohls et al., 2009). Thus, we measured children's reactivity to positive and negative motivational stimuli in the present study and expected children with greater reward sensitivity to show stronger effects of reward on behavioral performance.

## 2 METHODS

### 2.1 Participants

The participants were 25 healthy, typically developing, right‐handed Japanese preschoolers, who did not have any known developmental abnormalities (*M* age = 66.7 months, *SD* = 3.2, range = 60–72 months; 13 girls). All the participants were recruited from the Kyoto University database of parents who agreed to participate in child development studies. The database included the families who lived in Japan's Kansai area. Parents were invited to participate in the experiment by telephone. At this step, as reported by their parents, participants, who were left‐handed, had history of developmental disabilities or psychiatric conditions or were taking any medications for psychiatric of neurological conditions were excluded. We obtained written informed consent from them after explaining the content and the methods of the study at the examination room on the day of experiment. The study was conducted in accordance with the principles of the Declaration of Helsinki, approved by the Research Ethics Review Board at Kyoto University. Regarding all mothers’ education, 4% reported not completing high school, 12% completing high school, 24% completing some college, 44% completing a bachelor's degree, and the remaining 16% completing a graduate school degree. For all fathers’ education, 4% reported not completing high school, 8% completing high school, 4% completing some college, 64% completing a bachelor's degree, 16% completing a graduate school degree, and the remaining 4% missing data. These data were collected from the parents who did presented with their children.

### 2.2 Measures

#### 2.2.1 Motivation check scale

Although the rewards used in this study may increase children's extrinsic motivation, they could possibly undermine children intrinsic motivation (Deci, Koestner, & Ryan, 1999). The inconsistency of intrinsic motivation may affect children's performance (Carlton & Winsler, 1998). Therefore, children's intrinsic motivation was measured throughout the experiment for four times (i.e., before the start of the experiment and each condition) by using the motivation check scale. The experimenter showed children a set of five laminated cartoon character cards, representing how much he/she wants to perform the task (Qu et al., 2013). The gestures of the cartoon character show the distance between the two arms change from wide open (I really, really want to play the game) to gradually closing together until, ultimately, they are shown crossed (I do not want to play the game at all). Children were asked to point to the character that most closely corresponded to how much they desired to perform the task at the moment. Their response was scored using 5‐point scales (1 = really, really want; 2 = really want; 3 = want only a little bit; 4 = want it only a tiny bit; 5 = do not want at all).

#### 2.2.2 BIS/BAS

The personality trait measures of BIS and BAS functioning were used in this study (Blair, Peters, & Granger, 2004; Carver & White, 1994). We modified Takahashi et al. (2007) Japanese version of BIS/BAS scales by changing the pronouns (e.g., “I” to “My child”) for parent report. The BIS/BAS scales consist of 20 items. All items were evaluated by a Japanese expert in child development for accuracy and reliability and were checked whether the parents could understand all questions during the pilot study. The BIS is utilized as an indicator of withdrawal tendencies and is sensitive to signals of nonreward and punishment. Conversely, the BAS is utilized as an indicator of approach tendencies and it is sensitive to signals of reward and nonpunishment. The items, albeit presented in random order, fell into four categories corresponding to the BIS and three aspects of BAS (i.e., reward responsiveness, drive, and fun seeking). To maximize internal consistency, we removed one item from the BIS (i.e., “My child has very few fears as compared to his/her friends”), and the remaining six items were used for final analyses. The Cronbach's alphas were 0.74 for BIS (6 items), 0.68 for reward responsiveness (5 items), 0.80 for drive (4 items), 0.77 for fun seeking (4 items), and 0.87 for a combination of all BAS scale items (13 items). Each item was scored on a grading scale of 1 (extremely untrue of the child) to 7 (extremely true of the child). The average scores for BIS and BAS scales were calculated from the means of the scale items. The previous literature reported that the difference scores exhibited higher test–retest stability than did the scale scores (Sutton & Davidson, 1997). Hence, a BIS/BAS difference score was calculated by subtracting the *z*‐transformed BIS score from the *z*‐transformed BAS score for further analyses (Kohls et al., 2009). A positive difference score indicates relatively greater approach tendencies to incentives.

#### 2.2.3 Incentive Go/No‐go task

**Figure 1:**
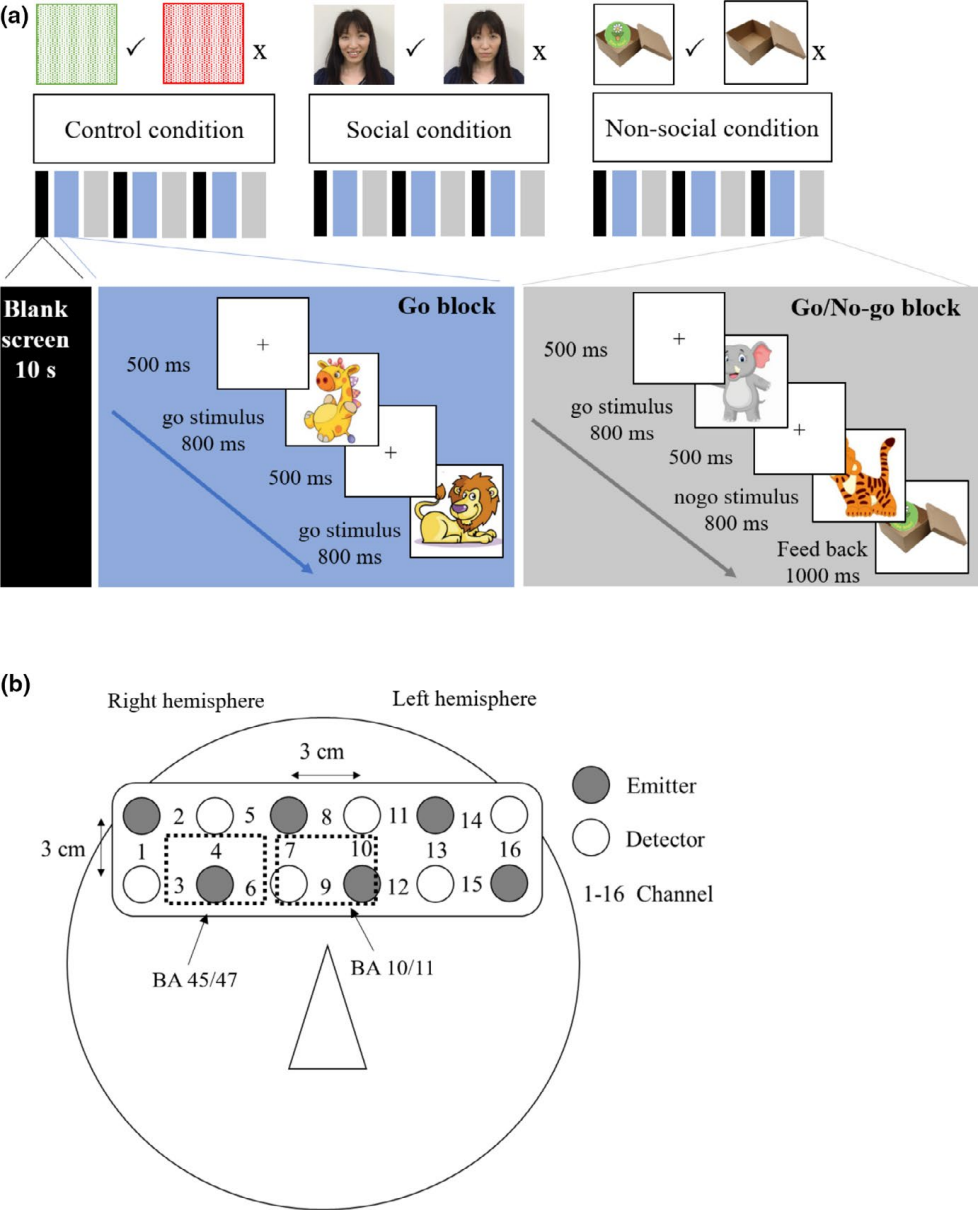
Experimental settings. (a) The procedure for the incentive Go/No‐go task. (b) The multichannel NIRS probe was attached to child's forehead in order to assess the frontal activity. The triangle represents child's nose. The region of interest included channels 3, 4, 6, 7, 9, and 10, which correspond to the right inferior frontal gyrus and the medial frontal gyrus

In this experiment, the incentive Go/No‐go task was created using PsychoPy v1.90.3 (https://www.psychopy.org). All children performed three conditions—control, social, and nonsocial reward. The order of conditions was counterbalanced across children. The procedure for our incentive Go/No‐go task, as shown in Figure 1a, was adapted from Monden et al. (2015). Each condition consisted of six blocks containing alternating Go and Go/No‐go blocks. In the Go block, the giraffe and lion cartoon pictures served as the go stimuli. Each Go block included 10 s for the blank screen, 3 s for the short instruction (e.g., This is the Go game), and 15 consecutive trials (9 giraffes and 6 lions with a pseudorandom order), respectively. In the Go/No‐go block, the elephant cartoon picture served as the go stimulus while the tiger cartoon picture served as the no‐go stimulus. Each Go/No‐go block included 3 s for the short instruction (e.g., This is the No‐go game) and 15 consecutive trials (9 elephants and 6 tigers with a pseudorandom order). Among the conditions and the practice session, the stimuli were the same animal cartoon characters but with different postures in order to avoid the habituation effect. The stimuli were presented in the center of the computer screen for 800 ms with a fixation of 500 ms. Children were asked to press a button for go stimuli as quickly as they could and to provide no response for no‐go stimuli. Only no‐go trials were followed by feedback pictures for 1,000 ms. The feedback types were adapted from Kohls et al. (2009). In the control condition, we used meaningless images in which green mosaic pictures served as positive reinforcers and red mosaic pictures were shown after false alarms. In the social reward condition, we used children's own parents’ images as feedback by taking the photographs of happy and neutral facial expressions on the day of the experiment. The happy facial expressions were shown after correct responses, while the neutral expressions were shown after false alarms. Some might argue that neutral expressions could be weaker social nonrewards in its emotional intensity and valence of the expression, relative to negative expressions (e.g., sad or angry faces). Although the neural sensitivity to positive versus negative feedbacks could have yielded stronger differences (Brunia, Hackley, van Boxtel, Kotani, & Ohgami, 2011), it would have been difficult to know whether those differences were mostly driven by the positive or negative feedback. In contrast, the comparison of positive and neutral feedbacks has a more straightforward interpretation. Our study was mostly interested in the influence of positive feedback or reward (not negative feedback or punishment). Hence, we decided to use neutral facial expression as social nonrewards instead of negative expressions. In the nonsocial reward condition, we used pictures of a box containing a sticker as the positive incentive and an empty box as the feedback for false alarms. The real stickers with the same amount of correct rejection were provided to children after the experiment. All stimuli and feedback pictures were on a white background and were equal in size. The accuracy in go trials and false alarm rates were calculated in percentage. Only response time for correct responses and higher than 200 ms were analyzed.

#### 2.2.4 NIRS recording system

We used multichannel NIRS equipment, operating at wavelengths of 770 and 840 nm (OEG‐16; Spectratech Inc.), to measure the relative changes in oxygenated hemoglobin (oxy‐Hb) and deoxygenated hemoglobin (deoxy‐Hb) during the incentive Go/No‐go task (see Figure 1b). The head module consisted of six light emitters and six light detectors arranged alternatively at an inter‐optode distance of 3 cm. The center of the headband was placed at Fpz in accordance with the International 10–20 system. The temporal resolution at each channel was approximately 82 ms. In our study, the region of interest (ROI) included channels 3, 4, 6, 7, 9, and 10, which correspond to the right IFG and the MFC (Moriguchi et al., 2018; Oboshi et al., 2014).

We analyzed NIRS data using OEG‐16 software V3.0 (Spectratech Inc.) and Python 2.7.13 (https://www.python.org/). First, we checked motion artifacts using video recordings of the sessions, which only revealed minor motions warranting no data rejection at that stage. Next, we removed 1% of data that were higher or lower than three standard deviations away from their mean. Then, we preprocessed individual data for the NIRS signal of each channel with a linear fitting and band‐pass filter (0.01–0.1 Hz) to minimize high‐ and low‐frequency noise (Yasumura et al., 2012). The NIRS signal was separated into functional signals (i.e., brain activation) and systematic signals (i.e., physiological artifacts). Our approach was based on a negative or positive linear relationship between oxy‐Hb and deoxy‐Hb changes (Yamada, Umeyama, & Matsuda, 2012). This method utilizes the known characteristics of NIRS signals in that oxy‐Hb and deoxy‐Hb negatively correlate in the case of cerebral function, whereas oxy‐Hb and deoxy‐Hb are positively correlated in the case of systemic function. Only the functional signals were used for our analyses (Moriguchi & Shinohara, 2018; Yamada et al., 2012). Generally, block designs tend to be efficient for assessing the signal magnitude differences between conditions and providing an adequate signal‐to‐noise ratio and a high statistical power (Herold, Wiegel, Scholkmann, & Müller, 2018). Hence, we used a block design for the Go/No‐go task in which the Go/No‐go block (target) was designed to induce inhibition while the Go block (baseline) was designed to control for the activation elicited by the motor responses. The time courses of oxy‐Hb and deoxy‐Hb signals were averaged over the three Go blocks and three Go/No‐go blocks. The data of hemodynamic responses for the short instruction (3 s preceding the task) were excluded (Monden et al., 2015). Then, the value of the new starting point was subtracted from each time point during the Go and the Go/No‐go block for baseline correction. For statistical analyses, we then averaged the oxy‐Hb and deoxy‐Hb responses for all time points (between 4 s after block onset and end of the block) of the Go and the Go/No‐go block for each condition, each channel, and each subject. This procedure yielded one value for the Go block and one for the Go/No‐go block of each condition, each channel, and each subject. We used these values for the main data analysis. Furthermore, for correlation analysis, we computed the difference in oxy‐Hb changes between the Go/No‐go block and the Go block by subtracting the mean oxy‐Hb changes of Go block from Go/No‐go block. The values represented the brain responses that deducted the effect of the motor responses.

### 2.3 Procedure

After we obtained their written informed consent, the parents were asked to complete the BIS/BAS scales in the designated area behind the curtain but in the same room with the child. The experiment was conducted by two experimenters. Experimenter 1 (E1) was appointed as a partner for the child. The child was assessed for intrinsic motivation using the motivation check scale before the start of the experiment. Then, E1 explained the instructions for the incentive Go/No‐go task, using laminated pictures, to the child. Experimenter 2 (E2) asked the child to wear the NIRS probe, and the practice session was started. The child performed four practice trials to ensure his/her understanding of the instructions. The child was allowed to repeat the practice trials if needed. Each child performed three reward conditions (i.e., control, social, and nonsocial). Before each condition, E1 assessed the child's intrinsic motivation again and informed the child about the feedback before starting the task. For nonsocial reward condition, children were informed in advance that they would receive one sticker for every correct rejection. All of the instructions were read in Japanese. The NIRS probe was removed by E2 when the child completed the task or declined to continue the experiment. The child received the stickers with the same amount of correct rejection in the nonsocial reward condition as a gift. The whole experimental procedure lasted about 45 min.

## 3 RESULTS

### 3.1 Motivation check scale

Since data deviated from normality, we used a nonparametric Friedman test of differences among repeated measures to determine whether the intrinsic motivation differed between time points. All children's intrinsic motivation was assessed four times, before starting the experiment and before each condition (1st: *M* = 1.64, *SD* = 0.95; 2nd: *M* = 1.68, *SD* = 0.99; 3rd: *M* = 2.20, *SD* = 1.19; 4th: *M* = 1.96, *SD* = 1.17), respectively. There were no significant differences in intrinsic motivation across assessments, *χ* ^2^(3) = 5.36, *p* = .15.

### 3.2 BIS/BAS

In Blair et al. (2004) study, their study examined a parent‐report version of the BIS/BAS scales in 170 preschoolers between the ages of 3 and 5 years. The average score of BIS was 30.78 (*SD* = 6.67), and the average score of BAS was 58.56 (*SD* = 7.42). However, these scores were equivalently converted to 3.60 (*SD* = 0.95) for BIS and 3.50 (*SD* = 0.57) for BAS in our scale.

In our study, the average score of BIS was 4.17 (*SD* = 1.03). The average scores of the three aspects of BAS (reward responsiveness, drive, and fun seeking) were 5.93 (*SD* = 0.80), 5.07 (*SD* = 1.00), and 5.21 (*SD* = 1.06), respectively. It resulted in a total score of BAS of 5.44 (*SD* = 0.80).

### 3.3 Incentive Go/No‐go task

#### 3.3.1 Behavioral data

All children completed the task. However, one child made errors by responding with the wrong button during the social reward condition. Thus, we excluded the data of that child, and the performance results were based on the data of the remaining 24 children. Notably, in the social condition, twenty‐three children saw their mother's images and two children saw their father's images. The descriptive data of incentive Go/No‐go performance were shown in Table 1.

**Table 1:** Summary of analyses of incentive Go/No‐go task performance by condition

Variable	*n*		Control	Social	Nonsocial	Statistics
Hits (%; Go block)	24	min.	62.22	48.89	40.00	*F*_(2, 46)_ = 0.13, *p = *.88, *ƞ* ^2^ = 0.01
		max.	100.00	100.00	100.00	
		*M*	82.04	82.13	83.43	
		*SD*	12.12	12.68	17.23	
Hits (%; Go/No‐go block)	24	min.	33.33	29.63	37.04	*F*_(2, 46)_ = 0.28, *p = *.76, *ƞ* ^2^ = 0.01
		max.	96.30	100.00	100.00	
		*M*	73.61	75.77	75.31	
		*SD*	18.77	18.66	18.65	
FA (%; Go/No‐go block)	24	min.	0.00	0.00	0.00	*χ* ^2^(2) = 0.03, *p* = .99
		max.	33.33	22.22	22.22	
		*M*	6.71	6.25	5.56	
		*SD*	8.02	7.20	5.67	
RT (ms; Go block)	24	min.	282.36	345.07	303.97	*F*_(2, 46)_ = 1.25, *p = *.30, *ƞ* ^2^ = 0.05
		max.	611.39	673.80	600.96	
		*M*	445.00	473.55	463.44	
		*SD*	86.53	92.27	78.18	
RT (ms; Go/No‐go block)	24	min.	420.80	441.25	438.74	*F*_(1.60, 36.90)_ = 0.004, *p = *.99, *ƞ* ^2^ < 0.001
		max.	658.11	661.33	652.14	
		*M*	558.76	559.33	558.25	
		*SD*	68.08	62.12	50.16	

Abbreviations: FA, false alarm; M, mean; RT, response time; *SD*, standard deviation.

We used a one‐way repeated‐measures ANOVA to determine the effects of the reward condition on task performance (i.e., hits and response time) in both the Go and Go/No‐go block. The alpha level was set at 0.05. However, there were no significant differences in hits (*F*(2, 46) = 0.13, *p* = .88, *ƞ* ^2^ = 0.01) and response time (*F*(2, 46) = 1.25, *p* = .30, *ƞ* ^2^ = 0.05) in the Go block and hits (*F*(2, 46) = 0.28, *p* = .76, *ƞ* ^2^ = 0.01) and response time (*F*(1.60, 36.90) = 0.004, *p* = .99, *ƞ* ^2^ < 0.001) in the Go/No‐go block across conditions (Table 1). Meanwhile, as the false alarms data had marked deviations from normality, a nonparametric Friedman test of differences among repeated measures was also conducted. Likewise, there was no statistically significant difference in false alarms across conditions (*χ* ^2^(2) = 0.03, *p* = .99). Since the parent's gender may differentially impact on children's performance, we focused on the children who saw the mother's face. A one‐way repeated‐measures ANOVA revealed no significant differences in hits (*F*(2, 42) = 0.88, *p* = .43, *ƞ* ^2^ = 0.04) and response time (*F*(2, 42) = 1.07, *p* = .35, *ƞ* ^2^ = 0.05) in the Go block and hits (*F*(2, 42) = 0.21, *p* = .82, *ƞ* ^2^ = 0.01) and response time (*F*(2, 42) = 0.01, *p* = .99, *ƞ* ^2^ < 0.001) in the Go/No‐go block across conditions. Also, a nonparametric Friedman test of differences among repeated measures showed no statistically significant difference in false alarms across conditions (*χ* ^2^(2) = 0.68, *p* = .71; Table S1).

In addition, we used mixed ANOVA to determine whether any change in task performance (i.e., hits, false alarms, and response times) is the result of the interaction between the reward condition and gender. However, no statistically significant interaction was found in hits (*F*(2, 44) = 0.43, *p* = .66, *ƞ* ^2^ = 0.02) and response time (*F*(2, 44) = 0.38, *p* = .68, *ƞ* ^2^ = 0.02) in the Go block and hits (*F*(2, 44) = 0.31, *p* = .73, *ƞ* ^2^ = 0.01), false alarms (*F*(2, 44) = 0.01, *p* = .99, *ƞ* ^2^ = 0.001), and response time (*F*(2, 44) = 0.69, *p* = .51, *ƞ* ^2^ = 0.03) in the Go/No‐go block (Table S2).

#### 3.3.2 NIRS data

In terms of the NIRS analyses, we examined prefrontal activation related to response inhibition during the Go/No‐go block compared to the Go block and investigated it varied across incentive types. A two‐way repeated‐measures ANOVA was applied to test the main effect of reward condition and block, and the interaction effect of reward condition and block on the oxy‐Hb changes in ROI. The reward condition included three levels (control, social, and nonsocial) and the block consisted of two levels (Go, Go/No‐go). We applied a 0.008 (0.05/6) alpha level of significance (six channels) for multiple comparisons.

**Figure 2:**
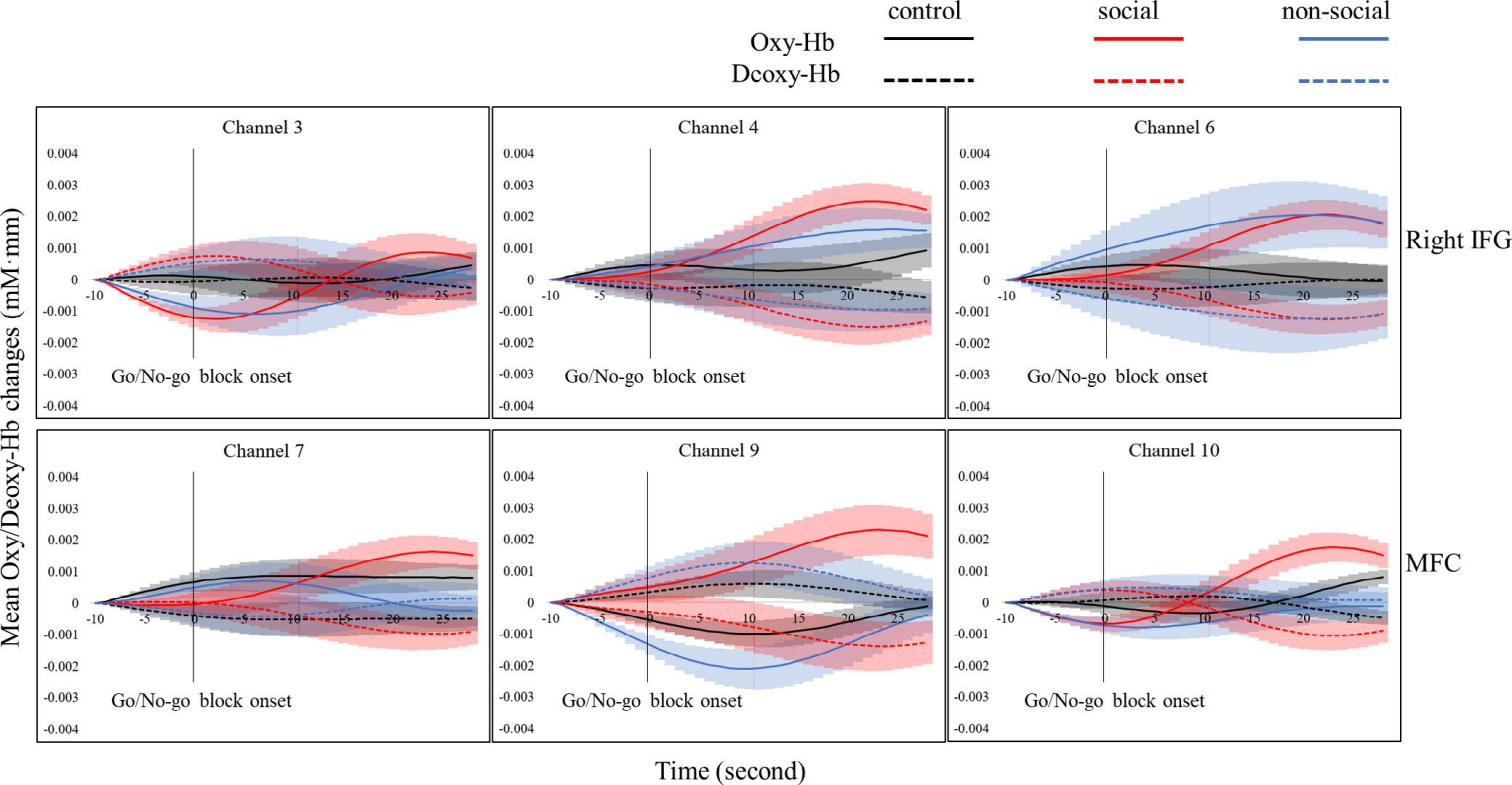
Temporal changes in the oxy‐Hb and deoxy‐Hb concentration in the right inferior frontal gyrus (right IFG; channels 3, 4, and 6) and the medial frontal gyrus (MFG; channels 7, 9, and 10) during the incentive Go/No‐go task. The grand‐average signals across the participants in each condition (color lines) and the standard error of the mean (color bands) were shown. The Go/No‐go block (target) was designed to induce inhibition. the Go block (baseline) was designed to control for the activation elicited by the motor responses. The black vertical line represents the point of Go/No‐go block onset

**Figure 3:**
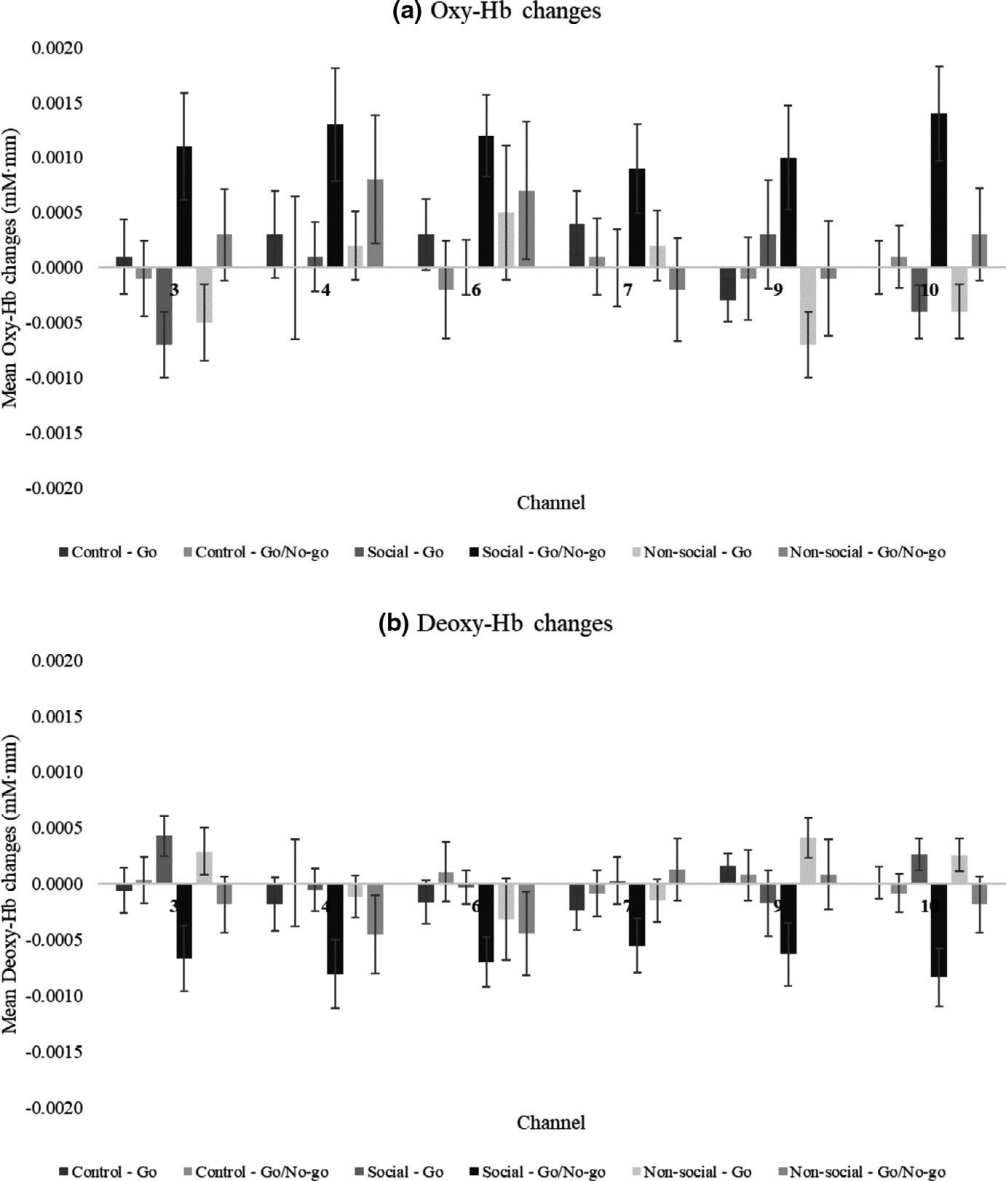
Mean (a) oxy‐Hb and (b) deoxy‐Hb changes separated by condition and block in channel 3, 4, 6, 7, 9, and 10. Error bars indicate the standard errors

We assessed whether oxy‐Hb changes in each channel differed as a function of the reward condition and block (Table 2 and Figures 2 and 3). A significant difference was found in channel 3 only. For channel 3, we did not find a main effect of reward condition (*F*(2,48) = 0.31; *p* = .74, corrected for multiple comparisons; *ƞ* ^2^ = 0.10). However, we found a main effect of block (*F*(1,24) = 11.88; *p* = .002, corrected for multiple comparisons; *ƞ* ^2^ = 0.33) and the interaction effect between reward and block (*F*(2,48) = 6.82; *p* = .002, corrected for multiple comparisons; *ƞ* ^2^ = 0.22). Post hoc tests using the Bonferroni correction revealed that oxy‐Hb changes during Go/No‐go block (*M* = 0.0011, *SD* = 0.0024) were higher than during Go block (*M* = −0.0007, *SD* = 0.0015, *p* < .001, *d* = 0.90) in the social reward condition, and the oxy‐Hb changes during Go/No‐go block (*M* = 0.0003, *SD* = 0.0021) were higher than during Go block (*M* = −0.0005, *SD* = 0.0017, *p* = .03, *d* = 0.42) in the nonsocial reward condition, but no significant differences between Go/No‐go block (*M* = −0.0001, *SD* = 0.0017) and Go block (*M* = 0.0001, *SD* = 0.0017, *p* = .69) were found in the control condition. Moreover, the oxy‐Hb changes in the social reward condition were significantly greater than in the control condition (*p* = .04, *d* = 0.58) in the Go/No‐go block, but not in the Go block (*p* = .16, *d* = 0.09). In this study, we also computed the difference in oxy‐Hb changes between the Go/No‐go block and the Go block. We used a one‐way repeated‐measures ANOVA to determine the effects of the reward conditions on the difference in oxy‐Hb changes between the Go/No‐go block and the Go block. The results showed patterns similar to the main results (Tables S3 and S4).

**Table 2:** Summary of analyses of oxy‐Hb changes by condition

Channel	*n*		Control		Social		Nonsocial		ANOVA		
			Go	Go/No‐go	Go	Go/No‐go	Go	Go/No‐go	Condition	Block	Interaction
3	25	min.	−0.0042	−0.0033	−0.0042	−0.0035	−0.0055	−0.0042	*F*_(2, 48)_ = 0.31,* p* = .74,* ƞ* ^2^ = 0.01	*F*_(1, 24)_ = 11.88, *p* = .002,* ƞ* ^2^ = 0.33	*F*_(2, 48)_ = 6.82, *p = *.002,* ƞ* ^2^ = 0.22
		max.	0.0042	0.0030	0.0024	0.0057	0.0040	0.0040			
		*M*	0.0001	−0.0001	−0.0007	0.0011	−0.0005	0.0003			
		*SD*	0.0017	0.0017	0.0015	0.0024	0.0017	0.0021			
4	25	min.	−0.0036	−0.0109	−0.0051	−0.0038	−0.0038	−0.0033	*F*_(2, 48)_ = 1.06, *p = *.35,* ƞ* ^2^ = 0.04	*F*_(1, 24)_ = 1.28, *p = *.27,* ƞ* ^2^ = 0.05	*F*_(2, 48)_ = 1.61, *p = *.21,* ƞ* ^2^ = 0.06
		max.	0.0051	0.0054	0.0024	0.0064	0.0035	0.0073			
		*M*	0.0003	0.0000	0.0001	0.0013	0.0002	0.0008			
		*SD*	0.0020	0.0033	0.0016	0.0026	0.0016	0.0029			
6	25	min.	−0.0038	−0.0056	−0.0030	−0.0024	−0.0030	−0.0059	*F*_(1.56, 37.32)_ = 0.92, *p = *.39,* ƞ* ^2^ = 0.04	*F*_(1,24)_ = 0.59, *p = *.45,* ƞ* ^2^ = 0.02	*F*_(2, 48)_ = 4.01, *p = *.03,* ƞ* ^2^ = 0.14
		max.	0.0052	0.0043	0.0027	0.0043	0.0134	0.0084			
		*M*	0.0003	−0.0002	0.0000	0.0012	0.0005	0.0007			
		*SD*	0.0016	0.0022	0.0013	0.0019	0.0031	0.0031			
7	23	min.	−0.0017	−0.0026	−0.0024	−0.0027	−0.0029	−0.0043	*F*_(2, 44)_ = 0.58, *p = *.57,* ƞ* ^2^ = 0.03	*F*_(1, 22)_ = 0.08, *p = *.78,* ƞ* ^2^ = 0.004	*F*_(2, 44)_ = 3.45, *p = *.04,* ƞ* ^2^ = 0.14
		max.	0.0048	0.0039	0.0045	0.0050	0.0041	0.0038			
		*M*	0.0004	0.0001	0.0000	0.0009	0.0002	−0.0002			
		*SD*	0.0014	0.0017	0.0017	0.0019	0.0015	0.0023			
9	25	min.	−0.0028	−0.0046	−0.0013	−0.0023	−0.0064	−0.0084	*F*_(2, 48)_ = 3.02, *p = *.06,* ƞ* ^2^ = 0.11	*F*_(1, 24)_ = 3.34, *p = *.08,* ƞ* ^2^ = 0.12	*F*_(2, 48)_ = 0.59, *p = *.56,* ƞ* ^2^ = 0.02
		max.	0.0016	0.0046	0.0112	0.0075	0.0016	0.0048			
		*M*	−0.0003	−0.0001	0.0003	0.0010	−0.0007	−0.0001			
		*SD*	0.0009	0.0019	0.0025	0.0024	0.0015	0.0026			
10	24	min.	−0.0031	−0.0027	−0.0033	−0.0033	−0.0030	−0.0042	*F*_(2, 46)_ = 1.63, *p = *.21,* ƞ* ^2^ = 0.07	*F*_(1, 23)_ = 9.96, *p = *.004,* ƞ* ^2^ = 0.30	*F*_(2, 46)_ = 3.82, *p = *.03,* ƞ* ^2^ = 0.14
		max.	0.0028	0.0022	0.0017	0.0058	0.0020	0.0037			
		*M*	0.0000	0.0001	−0.0004	0.0014	−0.0004	0.0003			
		*SD*	0.0012	0.0014	0.0012	0.0021	0.0012	0.0020			

Abbreviations: M, mean; *SD*, standard deviation.

In terms of deoxy‐Hb changes in each channel, a significant difference was also found in channel 3 only (Table 3 and Figures 2 and 3). For channel 3, we did not find a main effect of reward condition (*F*(2,48) = 0.31; *p* = .74, corrected for multiple comparisons; *ƞ* ^2^ = 0.10). However, we found a main effect of block (*F*(1,24) = 11.88; *p* = .002, corrected for multiple comparisons; *ƞ* ^2^ = 0.33) and the interaction effect between reward and block (*F*(2,48) = 6.82; *p* = .002, corrected for multiple comparisons; *ƞ* ^2^ = 0.22). Post hoc tests using the Bonferroni correction revealed that deoxy‐Hb changes during Go/No‐go block (*M* = −0.0007, *SD* = 0.0015) were lower than during Go block (*M* = 0.0004, *SD* = 0.0009, *p* < .001, *d* = 0.89) in the social reward condition, and the deoxy‐Hb changes during Go/No‐go block (*M* = −0.0002, *SD* = 0.0013) were lower than during Go block (*M* = 0.0003, *SD* = 0.0010, *p* = .03, *d* = 0.43) in the nonsocial reward condition, but no significant differences between Go/No‐go block (*M* = −0.0000, *SD* = 0.0010) and Go block (*M* = −0.0001, *SD* = 0.0010, *p* = .1) were found in the control condition. Moreover, the deoxy‐Hb changes in the social reward condition were significantly lower than in the control condition (*p* = .04, *d* = 0.55) in the Go/No‐go block, but not in the Go block (*p* = .16, *d* = 0.53).

**Table 3:** Summary of analyses of deoxy‐Hb changes by condition

Channel	*n*		Control			Social			Nonsocial		ANOVA		
			Go	Go/No‐go		Go	Go/No‐go		Go	Go/No‐go	Condition	Block	Interaction
3	25	min.	−0.0025	−0.0018	−0.0014	−0.0034	−0.0024	−0.0024	−0.0025	−0.0018	*F*_(2, 48)_ = 0.31,* p = *.74,* ƞ* ^2^ = 0.01	*F*_(1, 24)_ = 11.88, *p = *.002,* ƞ* ^2^ = 0.33	*F*_(2, 48)_ = 6.82, *p = *.002,* ƞ* ^2^ = 0.22
		max.	0.0025	0.0020	0.0025	0.0021	0.0033	0.0025	0.0025	0.0020			
		*M*	−0.0001	0.0000	0.0004	−0.0007	0.0003	−0.0002	−0.0001	0.0000			
		*SD*	0.0010	0.0010	0.0009	0.0015	0.0010	0.0013	0.0010	0.0010			
4	25	min.	−0.0030	−0.0032	−0.0014	−0.0038	−0.0021	−0.0044	−0.0030	−0.0032	*F*_(2, 48)_ = 1.06, *p = *.35,* ƞ* ^2^ = 0.04	*F*_(1, 24)_ = 1.28, *p = *.27,* ƞ* ^2^ = 0.05	*F*_(2, 48)_ = 1.61, *p = *.21,* ƞ* ^2^ = 0.06
		max.	0.0022	0.0065	0.0030	0.0023	0.0023	0.0020	0.0022	0.0065			
		*M*	−0.0002	0.0000	−0.0001	−0.0008	−0.0001	−0.0005	−0.0002	0.0000			
		*SD*	0.0012	0.0020	0.0010	0.0015	0.0009	0.0017	0.0012	0.0020			
6	25	min.	−0.0031	−0.0026	−0.0016	−0.0026	−0.0080	−0.0050	−0.0031	−0.0026	*F*_(1.56, 37.32)_ = 0.92, *p = *.39,* ƞ* ^2^ = 0.04	*F*_(1,24)_ = 0.59, *p = *.45,* ƞ* ^2^ = 0.02	*F*_(2, 48)_ = 4.01, *p = *.03,* ƞ* ^2^ = 0.14
		max.	0.0023	0.0034	0.0018	0.0014	0.0018	0.0035	0.0023	0.0034			
		*M*	−0.0002	0.0001	0.0000	−0.0007	−0.0003	−0.0004	−0.0002	0.0001			
		*SD*	0.0010	0.0013	0.0008	0.0011	0.0018	0.0019	0.0010	0.0013			
7	23	min.	−0.0029	−0.0024	−0.0027	−0.0030	−0.0024	−0.0023	−0.0029	−0.0024	*F*_(2, 44)_ = 0.58, *p = *.57,* ƞ* ^2^ = 0.03	*F*_(1, 22)_ = 0.08, *p = *.78,* ƞ* ^2^ = 0.004	*F*_(2, 44)_ = 3.45, *p = *.04,* ƞ* ^2^ = 0.14
		max.	0.0010	0.0016	0.0015	0.0016	0.0017	0.0026	0.0010	0.0016			
		*M*	−0.0002	−0.0001	0.0000	−0.0006	−0.0001	0.0001	−0.0002	−0.0001			
		*SD*	0.0009	0.0010	0.0010	0.0012	0.0009	0.0014	0.0009	0.0010			
9	25	min.	−0.0010	−0.0028	−0.0067	−0.0045	−0.0010	−0.0029	−0.0010	−0.0028	*F*_(2, 48)_ = 3.02, *p = *.06,* ƞ* ^2^ = 0.11	*F*_(1, 24)_ = 3.34, *p = *.08,* ƞ* ^2^ = 0.12	*F*_(2, 48)_ = 0.59, *p = *.56,* ƞ* ^2^ = 0.02
		max.	0.0017	0.0028	0.0008	0.0014	0.0038	0.0050	0.0017	0.0028			
		*M*	0.0002	0.0001	−0.0002	−0.0006	0.0004	0.0001	0.0002	0.0001			
		*SD*	0.0006	0.0011	0.0015	0.0014	0.0009	0.0016	0.0006	0.0011			
10	24	min.	−0.0017	−0.0013	−0.0010	−0.0035	−0.0012	−0.0022	−0.0017	−0.0013	*F*_(2, 46)_ = 1.63, *p = *.21, *ƞ* ^2^ = 0.07	*F*_(1, 23)_ = 9.96, *p = *.004,* ƞ* ^2^ = 0.30	*F*_(2, 46)_ = 3.82, *p = *.03,* ƞ* ^2^ = 0.14
		max.	0.0019	0.0016	0.0020	0.0020	0.0018	0.0025	0.0019	0.0016			
		*M*	0.0000	−0.0001	0.0003	−0.0008	0.0003	−0.0002	0.0000	−0.0001			
		*SD*	0.0007	0.0008	0.0007	0.0013	0.0007	0.0012	0.0007	0.0008			

Abbreviations: M, mean; *SD*, standard deviation.

Referring to these particular comparisons in both oxy‐Hb and deoxy‐Hb changes between social reward and control condition; however, it was not significant when fathers’ faces were excluded (Tables S5 and S6). No other significant differences between conditions were found in the Go block (*p*s > .05) in both oxy‐Hb and deoxy‐Hb changes.

In addition, we conducted a 3 (condition: control, social, nonsocial) × 2 (block: Go, Go/No‐go) × 2 (gender: boy, girl) ANOVA on the brain results. Again, our focus was interaction effects with gender, because gender may possibly influence the brain activity even if EF performance was equivalent. We applied a 0.008 (0.05/6) alpha level of significance (six channels) for multiple comparisons. As in the behavioral results, we found no significant two‐way interaction between condition and gender in all channels (channel 3, *F*(2, 46) = 0.95, *p* = .39, *ƞ* ^2^ = 0.04, channel 4, *F*(2, 46) = 0.03, *p* = .97, *ƞ* ^2^ = 0.001, channel 6, *F*(2, 46) = 1.19, *p* = .31, *ƞ* ^2^ = 0.50, channel 7, *F*(2, 42) = 0.16, *p* = .86, *ƞ* ^2^ = 0.01, channel 9, *F*(2, 44) = 0.98, *p* = .38, *ƞ* ^2^ = 0.04, and channel 10, *F*(2, 44) = 0.34, *p* = .72, *ƞ* ^2^ = 0.02, all *p*s were corrected for multiple comparisons). Further, we found no significant two‐way interaction between block and gender in all channels (channel 3, *F*(1, 23) = 0.66, *p* = .43, *ƞ* ^2^ = 0.03, channel 4, *F*(1, 23) = 0.92, *p* = .35, *ƞ* ^2^ = 0.04, channel 6, *F*(1, 23) = 0.55, *p* = .57, *ƞ* ^2^ = 0.03, channel 7, *F*(1, 21) = 1.59, *p* = .22, *ƞ* ^2^ = 0.07, channel 9, *F*(1, 22) = 1.80, *p* = .19, *ƞ* ^2^ = 0.08, and channel 10, *F*(1, 22) = 2.77, *p* = .11, *ƞ* ^2^ = 0.11, all *p*s were corrected for multiple comparisons). Importantly, the three‐way interaction between condition, block, and gender was not significant in any of the channels (channel 3, *F*(2, 46) = 1.75, *p* = .19, *ƞ* ^2^ = 0.07, channel 4, *F*(2, 46) = 0.75, *p* = .48, *ƞ* ^2^ = 0.03, channel 6, *F*(2, 46) = 0.03, *p* = .97, *ƞ* ^2^ = 0.001, channel 7, *F*(2, 42) = 1.76, *p* = .19, *ƞ* ^2^ = 0.08, channel 9, *F*(2, 44) = 0.56, *p* = .57, *ƞ* ^2^ = 0.03, and channel 10, *F*(2, 44) = 0.74, *p* = .48, *ƞ* ^2^ = 0.03, all *p*s were corrected for multiple comparisons; Table S7). In terms of deoxy‐Hb changes, the results were similar to the oxy‐Hb signal (Table S8).

### 3.4 Correlations across variables

In the current study, Pearson correlations were computed to examine the relationship (a) between task performance and the BIS/BAS and (b) between age, task performance (i.e., hits in Go/No‐go block), and difference in mean oxy‐Hb changes between the Go/No‐go block and the Go block.

**Figure 4:**
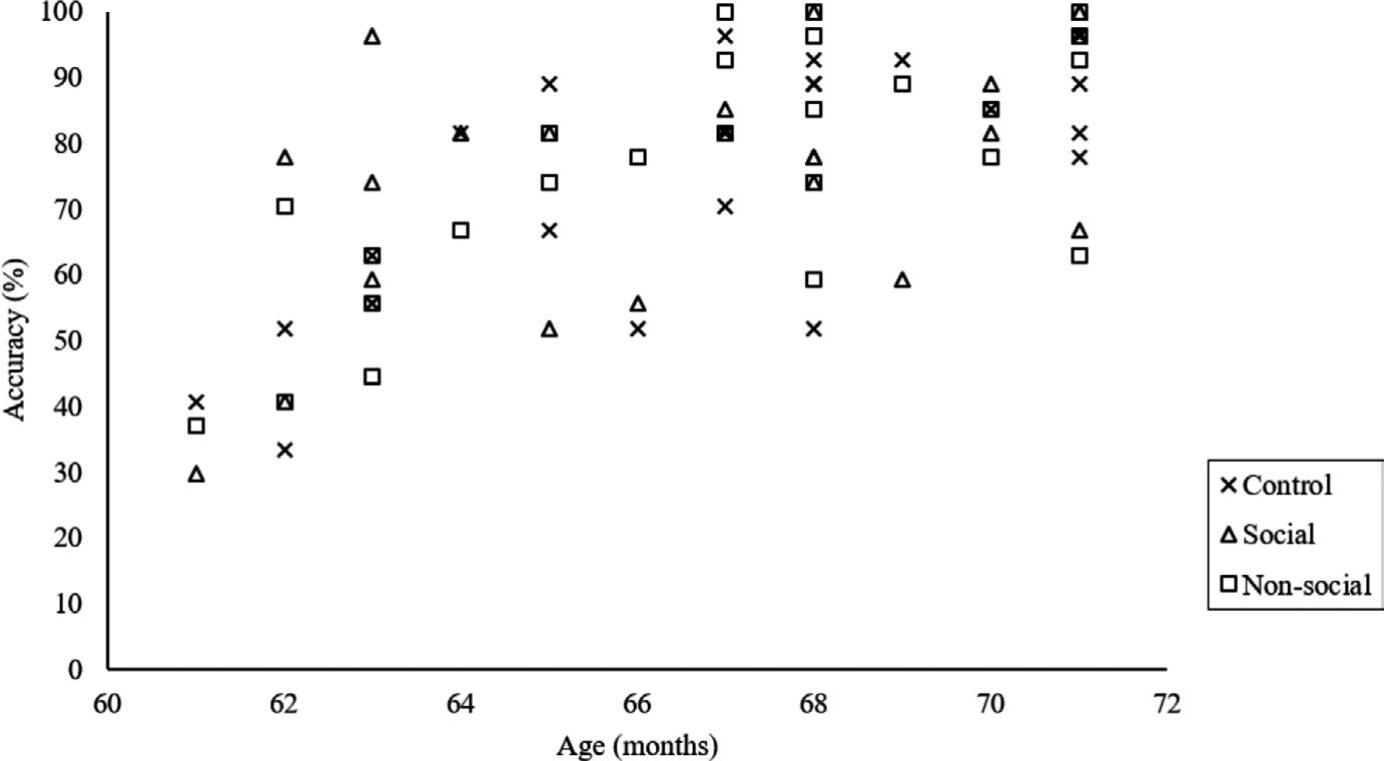
Scatter plot shows associations between age in month and percent accuracy of the hit rates in the Go/No‐go block for control, social, and nonsocial condition

Regarding the relationship between task performance and the BIS/BAS, we applied a 0.001 (0.05/36) alpha level of significance (36 correlations) for multiple comparisons. No other significant correlations were found between task performance and the BIS/BAS (*p*s > .001; Table 4). In terms of the relationship between age, task performance (i.e., hits in Go/No‐go block), and the difference in oxy‐Hb changes between the Go/No‐go block and the Go block, we applied a 0.001 (0.05/40) alpha level of significance (40 correlations) for multiple comparisons. For Bonferroni correction, we restricted the number of correlations run based on our hypotheses that social and nonsocial rewards would increase motivation in preschool children and promote greater EF behavioral performance. In total, we ran 40 correlations resulting in alpha = 0.001 (0.05/40), corresponding to correlations between (a) age and EF performance (4 correlations), (b) age and NIRS signal (18 correlations), and (c) EF performance and NIRS signal within the same condition (18 correlations). Consequently, we found that age was significantly and positively correlated with the hit rates in the Go/No‐go block for control, *r*(24) = 0.73, *p* < .001, and nonsocial reward condition, *r*(24) = 0.71, *p* < .001 (Table 5 and Figure 4). No other significant correlations were found between age and NIRS signal and between task performance and NIRS signal (*p*s > .001).

**Table 4:** Bivariate correlations between task performance and the BIS/BAS

Behavioral data in Go/No‐go block	BIS	Drive	Reward	Fun	BAS	BIS/BAS difference score
Hits						
Control	0.06	0.10	0.08	−0.19	0.00	−0.05
Social	0.17	0.06	0.11	−0.18	−0.01	−0.12
Nonsocial	0.03	0.02	0.02	−0.18	−0.06	−0.06
Reaction time						
Control	0.28	−0.15	−0.24	−0.32	−0.28	−0.39
Social	−0.05	0.08	0.14	0.03	0.10	0.10
Nonsocial	0.16	−0.05	0.05	−0.10	−0.04	−0.14

**Table 5:** Bivariate correlations between age, task performance, and the difference in oxy‐Hb changes between the Go/No‐go block and the Go block

Variable	Age	Behavioral data (Hits in Go/No‐go block)		
		Control	Social	Nonsocial
Age	1	0.73**	0.55	0.71**
NIRS data				
Control				
Ch3	−0.14	−0.30		
Ch4	−0.04	0.14		
Ch6	0.11	−0.13		
Ch7	−0.16	−0.18		
Ch9	0.00	−0.32		
Ch10	−0.02	0.03		
Social				
Ch3	0.25		0.22	
Ch4	0.00		0.01	
Ch6	0.15		0.01	
Ch7	0.05		0.23	
Ch9	−0.15		−0.13	
Ch10	0.23		0.21	
Nonsocial				
Ch3	0.01			−0.26
Ch4	0.05			−0.16
Ch6	0.35			−0.05
Ch7	0.09			−0.07
Ch9	0.30			−0.02
Ch10	0.08			−0.22

** Correlation is significant at the 0.01 level (2‐tailed), corrected for multiple comparison.

## 4 DISCUSSION

In the current study, we examined how social and nonsocial reward affects EF performance in typically developing 5‐ to 6‐year‐old children. Consistent with our hypothesis, the social reward condition enhanced right IFG activity (channel 3). In contrast to our hypothesis, however, we did not find any significant differences in children's motivation and EF performance based on reward conditions.

In line with our hypothesis, social reward promoted activation in right IFG. Previous studies claimed that the right IFG is functionally associated with action inhibition and execution and can be triggered by the EF tasks, such as Go/No‐go and stop signal task (Aron et al., 2014; Monden et al., 2015). Thus, our study implied that social reward could enhance activation in EF‐related brain area, which is rapidly developed in preschoolers. Social reward has been reported to be effective reinforcers during childhood (Demurie et al., 2012; Wang et al., 2017). Previous ERP study in children aged 6–8 years revealed an increased brain response in reward anticipation of faces, relative to nonface stimuli (Stavropoulos & Carver, 2014). Compared to the control condition, social reward using parents’ smiles in our study could have been especially rewarding and motivating for children. It is plausible that parents’ face may have satisfied children's innate psychological needs: the need for competence, relatedness, and autonomy (Deci, Vallerand, Pelletier, & Ryan, 1991). These basic needs are important for building up children's motivation, which may later affect children's EF engagement. In terms of neuronal mechanism, previous literature suggested various ways in which reward processing possibly influence PFC activity and thus EF engagement, including the transmission of dopamine in the mesolimbic and mesocortical pathway (Beierholm et al., 2013) and the connections of the PFC within itself and with other brain areas such as anterior cingulate cortex (Barbas & Pandya, 1989; Haber, Fudge, McFarland, 2000; Kouneiher et al., 2009; Shenhav et al., 2013). However, there is limited study in these aspects for preschool children, and they should be further investigated in detail.

In contrast to recent neuroimaging studies (Minagawa‐Kawai et al., 2009; O'Doherty et al., 2003), we did not find activation in the MFC (channel 7, 9, and 10), which is part of the reward‐related network, nor was it enhanced in social reward conditions. There are at least three plausible interpretations. First, the reward conditions possibly influence activation in other brain regions underlying the reward system, such as anterior cingulate cortex and striatum, which the NIRS device cannot detect. Therefore, other neuroimaging techniques (e.g., fMRI) should be implemented to gather more information about how social reward may influence activation in these other regions. Second, since our study focused on the preschool children who are a vulnerable population, thus, the present study did not employ any negative stimuli, especially in social reward, for incorrect responses. Comparing positive to negative reward may have yielded a greater difference in the neural signals (Brunia et al., 2011). This is similar to Stavropoulos and Carver’s (2014) ERP study, in which they did not observe larger stimulus preceding negativity (SPN), which involves neural response in reward anticipation, in the right hemisphere. They claimed that the SPN amplitude did not differ between hemispheres due to the absence of negative feedback. Therefore, future studies should carefully consider the use of incentive in reward and nonreward conditions in this age‐group. Next, the other plausible explanation for our results relates to the dissociation between reward processing/motivation and EF engagement in young children, that is, the internal connections of the prefrontal cortex (Barbas & Pandya, 1989; Kouneiher et al., 2009) and the corticostriatal reward network (Haber et al., 2000). Indeed, the PFC continues to develop until late adolescence (Kolb et al., 2012; Moriguchi & Hiraki, 2013). Similarly, the corticostriatal reward network is not fully developed until adulthood (Casey et al., 2008). Thus, the effect of motivation on EF, especially in the neuronal level, may not be observable yet in early childhood. As the interconnections among these networks increase during development, a dynamic interplay between PFC and the reward system may emerge later in childhood (Padmanabhan, Geier, Ordaz, Teslovich, & Luna, 2011; Van Leijenhorst et al., 2010). As a result, motivational interventions/activities may be more effective in engaging EF in older children than younger children. Hence, future investigations should include a broad age range from early childhood to young adults in order to examine the neurodevelopmental aspects of the brain circuitry underlying the processing of social and nonsocial reward across age‐groups.

Additionally, in contrast to previous studies in adolescence and adulthood (Knutson et al., 2001; Lin et al., 2012), our results showed that the nonsocial reward condition did not increase activation in the MFC. Unlike other studies, we used stickers as an incentive instead of financial items, since the concept of money is not completely understood by preschool age (Berti & Bombi, 1981; Grunberg & Anthony, 1980). Although stickers served as tangible reinforcers in many prior studies and are common in classroom contexts, they may not have been attractive enough to significantly increase MFC activation. Thus, other tangible rewards, such as toys or snacks, may yield greater brain responses in future studies.

In terms of EF performance, we did not find the motivational valence differences on task accuracy. This is similar to Beck et al. (2011) findings in which Beck and colleagues found the effect of age‐group but not the effect of affective‐motivational valence on preschoolers’ performance in the conflict task. It is notable that the average percentages of hits during the Go/No‐go blocks are relatively high (i.e., 73.61%–75.77%) and the average percentages of false alarms in our study are very low (i.e., 5.56%–6.7%). It possibly implies the floor and ceiling effects in this age, which may cause no difference on task accuracy across condition. However, our findings differed from those of Qu et al. (2013). Qu and colleagues found that the impact of reward anticipation on 4‐ to 5‐year‐old children's EF performance resulted in better performance in the Day/Night Stroop task when compared with the reward‐uninformed condition. By contrast, our study investigated the impact of reward provision on EF by presenting the reward as response feedback in No‐go trials, where we did not find differences in children's EF performance across reward conditions. Previous neuroimaging studies suggest that reward anticipation evokes different neuronal patterns relative to the receipt of reward outcomes (Knutson et al., 2001; Schultz, Tremblay, & Hollerman, 2000), which may account for the diverging findings across the two studies. Moreover, our results differed from Tarullo et al. (2018) study, where trial‐by‐trial reward feedback affected the accuracy of DCCS task in children aged 3.5–4.5 years. In Tarullo and colleagues’ study, each feedback included a combination of rewards; sound, facial expression, and stickers. The simultaneous use of various kinds of feedback probably has stronger effects on appetitive approach than the use of one kind of feedback at a time.

Furthermore, in our study, children's reward sensitivity measured by the parent‐reported BIS/BAS scale did not relate to cognitive control performance in any condition. By contrast, Kohls et al. (2009) reported that children with higher reward sensitivity showed greater improvements in cognitive control performance in the monetary reward condition but not in the social reward condition. They suggested that distinct temperamental traits influenced how task performance varied across the different reward conditions. Unlike Kohls et al.’s study, we used stickers instead of money as nonsocial reward. Stickers and money may convey different concepts in terms of their utilization. Children may not perceive stickers in the same way as adults perceive money, as unlike money, stickers are not exchangeable items. Hence, stickers may not provide the same benefit as money. However, since both items have never been directly compared, further investigation is needed.

The present results contribute to our understanding of cool and hot distinction of EF. Zelazo and colleagues proposed a framework of cool and hot distinction in EF (Zelazo & Carlson, 2012; Zelazo & Müller, 2002), where hot EF includes processes elicited under motivational contexts, whereas cool EF is utilized in neutral, nonaffective situations (Bunch & Andrews, 2012; Wilson, Andrews, Hogan, Wang, & Shum, 2018). Moreover, it has been proposed that the neural mechanisms may differ in cool and hot EF, where the lateral PFC and the anterior cingulate cortex play an important role in cool EF whereas orbitofrontal cortex may be related to hot EF (Zelazo & Müller, 2002). Previous studies classified research into cool or hot depending on the tasks being used. For example, a Go/No‐go task is generally regarded as tapping inhibition and cool EF and the delay of gratification is used as hot EF task. However, the hot and cool distinction is relative and both aspects may be involved in any given task at the behavioral and the neural level (Moriguchi & Shinohara, 2019; Zelazo & Müller, 2002). In the present study, the Go/No‐go task can be regarded as mainly tapping the cool EF task in the control condition whereas the reward conditions can be regarded as hot EF task. Moreover, in the social reward condition, the lateral PFC rather than MFC was significant activated. The neuroimaging data suggest that hot EF can recruit the lateral PFC, hence showing that hot and cool EF are supported by largely overlapping brain regions. This suggests that they form a single continuum rather than discrete forms of EF.

This study has some limitations that need to be considered. Regarding the data analyses, the sample size of this study was small which may cause a Type II error. In terms of NIRS analyses, it should be noted that the condition by block interaction in channel 6, 9, and 10 has medium effect size although the interaction did not reach significance level. The general pattern is consistent across channels, where activations in the Go/No‐go block were stronger than Go block in the social condition, but not in other conditions. Therefore, with greater statistical power, the interaction between block and condition may have turned significant in these channels too. Further, a differential path‐length factor may vary depending on children's age and can affect the results. Thus, the increasing of sample size and age‐specific differential path‐length factor value should be considered in future study. Regarding the experimental paradigm, we employed a block design as it is simpler to implement, statistically powerful, and straightforward to analyze (Herold et al., 2018). However, the individual trials cannot be compared with this design. More specifically, the Go/No‐go blocks in this study included both go and no‐go conditions. Due to the limitation on the synchronization between the computer for collecting behavioral data and the NIRS recording system, we could not analyze event‐related data of oxy‐Hb signal changes in this study. Thus, future works should consider to apply the event‐related design. Moreover, in this study, we judged the motion artifacts from video recordings which is subjective and could not remove all artifacts. Other methods or algorithms of measuring motion artifacts with objective threshold should be extended. Since this study examined the short‐term effects of reward motivation on EF, future developmental studies are required to investigate whether different incentive types could have differential long‐term effects.

## 5 CONCLUSION

In summary, this study expands our understanding of motivation and EF engagement in preschoolers. Specifically, social reward enhanced prefrontal activations in young children. Our results may have some noteworthy implications for parenting and classroom management, especially the use of age‐appropriate reinforcement to encourage children's behaviors. However, the reward conditions did not completely affect neural activity of reward processing, potentially because of immature connectivity between the neural circuits of these two systems in young children. Since we have not examined activity in striatum and other regions of the reward network that are out of reach, using NIRS, further analysis is necessary to validate this behavior. Detailed experiments with delicate constraints can be examined in order to understand the developments of these two systems in young children.

## CONFLICT OF INTEREST

The authors declare that they have no conflict of interest.

## AUTHOR CONTRIBUTION

KL and YM developed the study concept. All authors contributed to the study design. Data collection was performed by KL. The data analysis and interpretation were performed by all authors. KL and YM drafted the manuscript. All authors approved the final version of the manuscript for submission.

## ETHICAL APPROVAL

All parents were provided written legal guardian informed consent forms, and informed consent was obtained from all parents. The study protocol was approved by the Research Ethics Review Board of Kyoto University and was in accordance with the 1964 Helsinki declaration and its later amendments.

### Peer Review

The peer review history for this article is available at https://publons.com/publon/10.1002/brb3.1763.

## Supporting information

Supinfo

## Data Availability

The data that support the findings of this study are available from the corresponding author upon reasonable request.
