# Supplementary material for: Effects of social and nonsocial reward on executive function in preschoolers
Source: Brain Behav. 2020 Jul 30;10(9):e01763. doi: 10.1002/brb3.1763 (PMC7507562; doi:10.1002/brb3.1763)
Supplement: Supplementary file 1 — Supinfo [file BRB3-10-e01763-s001.docx]

**Supplementary Information**

We have reanalyzed both behavioral and NIRS data by excluding fathers’ faces from the entire sample. The results are similar to the main document. For NIRS, we also analyzed the effect of reward conditions on the difference in hemodynamic responses between the Go/No-go block and the Go block. However, the results showed the similar patterns to the main results. In addition, we examined the effect of children’s gender on both behavioral and brain results. The detailed analyses are presented in the following session.

**Incentive Go/No-go task**

**Behavioral data**

According to the parents’ gender, a one-way repeated measures ANOVA revealed no significant differences in hits (*F*(2, 42) = 0.88, *p* = .43, ƞ^2^ = 0.04) and response time (*F*(2, 42) = 1.07, *p* = .35, ƞ^2^ = 0.05) in the Go block and hits (*F*(2, 42) = 0.21, *p* = .82, ƞ^2^ = 0.01) and response time (*F*(2, 42) = 0.01, *p* = .99, ƞ^2^ < 0.001) in the Go/No-go block across conditions. Also, a non-parametric Friedman test of differences among repeated measures showed no statistically significant difference in false alarms across conditions, χ^2^(2) = 0.19, *p* = .91 (Table S1).

In addition, we used mixed ANOVA to determine whether any change in task performance (i.e., hits, false alarms, and response times) is the result of the interaction between the reward condition and gender. However, no statistically significant interaction was found in hits (*F*(2, 44) = 0.43, *p* = .66, ƞ^2^ = 0.02) and response time (*F*(2, 44) = 0.38, *p* = .68, ƞ^2^ = 0.02) in the Go block and hits (*F*(2, 44) = 0.31, *p* = .73, ƞ^2^ = 0.01), false alarms (*F*(2, 44) = 0.01, *p* = .99, ƞ^2^ = 0.001), and response time (*F*(2, 44) = 0.69, *p* = .51, ƞ^2^ = 0.03) in the Go/No-go block (Table S2).

**NIRS data**

In addition to the main analyses which were reported in the main manuscript, in this study, we also computed the difference in hemodynamic responses between the Go/No-go block and the Go block by subtracting the mean hemodynamic responses of Go block from Go/No-go block for each condition, each channel, and each subject. The values represented the brain responses that deducted the effect of the motor responses. In terms of oxy-Hb changes, a one-way repeated measures ANOVA was conducted to compare the effect of reward condition on the difference in oxy-Hb responses between the Go/No-go block and the Go block in each channel. The results revealed a significant effect of the reward condition on oxy-Hb changes (*F*(2, 48) = 6.82, *p* = .002, ƞ^2^ = 0.22) in channel 3 only. Post-hoc tests using the Bonferroni correction revealed that the difference in oxy-Hb responses between the Go/No-go block and the Go block in the social reward condition (*M* = 0.0018, *SD* = 0.0022) were higher than in the control condition (*M* = -0.0002, *SD* = 0.0019, *p* = .004, *d* = 0.96). No significant differences between the difference in oxy-Hb responses between the Go/No-go block and the Go block in the nonsocial reward condition (*M* = 0.0008, *SD* = 0.0017) and in the control condition (*p* = .22) were found. Similarly, no significant differences between the difference in oxy-Hb responses between the Go/No-go block and the Go block in the nonsocial reward condition and in the social reward condition (*p* = .22) were found (Table S3). For deoxy-Hb changes, the results revealed a similar pattern. There was a significant effect of the reward condition on deoxy-Hb changes (*F*(2, 48) = 6.82, *p* = .002, ƞ^2^ = 0.22) in channel 3 only. Post-hoc tests using the Bonferroni correction revealed that the difference in deoxy-Hb responses between the Go/No-go block and the Go block in the social reward condition (*M* = -0.0011, *SD* = 0.0013) were lower than in the control condition (*M* = -0.0001, *SD* = 0.0011, *p* = .004, *d* = 0.96). No significant differences between the difference in deoxy-Hb responses between the Go/No-go block and the Go block in the nonsocial reward condition (*M* = -0.0005, *SD* = 0.0010) and in the control condition (*p* = .22) were found. Likewise, no significant differences between the difference in deoxy-Hb responses between the Go/No-go block and the Go block in the nonsocial reward condition and in the social reward condition (*p* = .22) were found (Table S4).

According to the parents’ gender, a two-way repeated measures ANOVA was applied to compare the main effect of reward condition (control, social, and nonsocial) and block (Go, Go/No-go), and the interaction effect of reward condition and block on the oxy-Hb changes in ROI. We applied a 0.008 (0.05/6) alpha level of significance (six channels) for multiple comparisons. The results revealed a significant difference in channel 3 only (Table S5). We did not find a main effect of reward condition (*F*(2,44) = 0.26; *p* = .74, corrected for multiple comparisons; ƞ^2^ = 0.01). However, we found a main effect of block (*F*(1,22) = 17.11; *p* < .001, corrected for multiple comparisons; ƞ^2^ = 0.44) and the interaction effect between reward and block (*F*(2,44) = 5.91; *p* = .005, corrected for multiple comparisons; ƞ^2^ = 0.21). Post-hoc tests using the Bonferroni correction revealed that oxy-Hb changes during Go/No-go block (*M* = 0.0013 , *SD* = 0.0024) were higher than during Go block (*M* = -0.0007, *SD* = 0.0015, *p* < .001, *d* = 1.00) in the social reward condition, and the oxy-Hb changes during Go/No-go block (*M* = 0.0004, *SD* = 0.0019) were higher than during Go block (*M* = -0.0004, *SD* = 0.0018, *p* = .03, *d* = 0.43) in the nonsocial reward condition, but no significant differences between Go/No-go block (*M* = 0.0001, *SD* = 0.0016) and Go block (*M* = 0.0001, *SD* = 0.0018, *p* = .94) were found in the control condition. No other significant differences between conditions were found in both Go block and Go/No-go block (*p*s > .05).

In terms of deoxy-Hb changes in each channel, a significant difference was also found in channel 3 only (Table S6). For channel 3, We did not find a main effect of reward condition (*F*(2,44) = 0.26; *p* =.74, corrected for multiple comparisons; ƞ^2^ = 0.01). However, we found a main effect of block (*F*(1,22) = 17.11; *p* < .001, corrected for multiple comparisons; ƞ^2^ = 0.44) and the interaction effect between reward and block (*F*(2,44) = 5.91; *p* = .005, corrected for multiple comparisons; ƞ^2^ = 0.21). Post-hoc tests using the Bonferroni correction revealed that deoxy-Hb changes during Go/No-go block (*M* = -0.0008 , *SD* = 0.0015) were lower than during Go block (*M* = 0.0004, *SD* = 0.0009, *p* < .001, *d* = 0.97) in the social reward condition, and the deoxy-Hb changes during Go/No-go block (*M* = -0.0003, *SD* = 0.0012) were lower than during Go block (*M* = 0.0002, *SD* = 0.0011, *p* = .03, *d* = 0.43) in the nonsocial reward condition, but no significant differences between Go/No-go block (*M* = -0.0001, *SD* = 0.0010) and Go block (*M* = -0.0001, *SD* = 0.0011, *p* = .94) were found in the control condition. No other significant differences between conditions were found in both Go block and Go/No-go block (*p*s > .05).

In addition, we conducted a 3 (condition: control, social, nonsocial) x 2 (block: Go, Go/No-go) x 2 (gender: boy, girl) ANOVA on the brain results. Again, our focus was interaction effects with gender, because gender may possibly influence the brain activity even if EF performance was equivalent. We applied a 0.008 (0.05/6) alpha level of significance (six channels) for multiple comparisons. As in the behavioral results, we found no significant two-way interaction between condition and gender in all channels (channel 3, *F*(2, 46) = 0.95, *p* = .39, ƞ^2^ = 0.04, channel 4, *F*(2, 46) = 0.03, *p* = .97, ƞ^2^ = 0.001, channel 6, *F*(2, 46) = 1.19, *p* = .31, ƞ^2^ = 0.50, channel 7, *F*(2, 42) = 0.16, *p* = .86, ƞ^2^ = 0.01, channel 9, *F*(2, 44) = 0.98, *p* = .38, ƞ^2^ = 0.04, and channel 10, *F*(2, 44) = 0.34, *p* = .72, ƞ^2^ = 0.02, all *p*s were corrected for multiple comparisons). Further, we found no significant two-way interaction between block and gender in all channels (channel 3, *F*(1, 23) = 0.66, *p* = .43, ƞ^2^ = 0.03, channel 4, *F*(1, 23) = 0.92, *p* = .35, ƞ^2^ = 0.04, channel 6, *F*(1, 23) = 0.55, *p* = .57, ƞ^2^ = 0.03, channel 7, *F*(1, 21) = 1.59, *p* = .22, ƞ^2^ = 0.07, channel 9, *F*(1, 22) = 1.80, *p* = .19, ƞ^2^ = 0.08, and channel 10, *F*(1, 22) = 2.77, *p* = .11, ƞ^2^ = 0.11, all *p*s were corrected for multiple comparisons). Importantly, the three-way interaction between condition, block, and gender was not significant in any of the channels (channel 3, *F*(2, 46) = 1.75, *p* = .19, ƞ^2^ = 0.07, channel 4, *F*(2, 46) = 0.75, *p* = .48, ƞ^2^ = 0.03, channel 6, *F*(2, 46) = 0.03, *p* = .97, ƞ^2^ = 0.001, channel 7, *F*(2, 42) = 1.76, *p* = .19, ƞ^2^ = 0.08, channel 9, *F*(2, 44) = 0.56, *p* = .57, ƞ^2^ = 0.03, and channel 10, *F*(2, 44) = 0.74, *p* = .48, ƞ^2^ = 0.03, all *p*s were corrected for multiple comparisons; Table S7). In terms of deoxy-Hb changes, the results were similar to the oxy-Hb signal (Table S8).

**Table S1** Summary of analyses of incentive Go/No-go task performance by condition when fathers’ faces were excluding

| **Variable** | ***n*** |  | **Control** | **Social** | **Nonsocial** | **Statistics** |
| --- | --- | --- | --- | --- | --- | --- |
| Hits (%; Go block) | 22 | min. | 64.44 | 60.00 | 48.89 | *F*_(2, 42)_ = 0.88, *p =* 0.43, ƞ^2^ = 0.04 |
|  |  | max. | 100.00 | 100.00 | 100.00 |  |
|  |  | *M* | 83.64 | 83.44 | 86.77 |  |
|  |  | *SD* | 11.32 | 10.99 | 13.43 |  |
| Hits (%; Go/No-go block) | 22 | min. | 33.33 | 29.63 | 37.04 | *F*_(2, 42)_ = 0.21, *p =* 0.82, ƞ^2^ = 0.01 |
|  |  | max. | 96.30 | 100.00 | 100.00 |  |
|  |  | *M* | 73.57 | 75.42 | 75.25 |  |
|  |  | *SD* | 18.41 | 19.48 | 18.83 |  |
| FA (%; Go/No-go block) | 22 | min. | 0.00 | 0.00 | 0.00 | χ^2^(2) = 0.68, *p* = 0.71 |
|  |  | max. | 33.33 | 22.22 | 22.22 |  |
|  |  | *M* | 7.07 | 5.56 | 5.05 |  |
|  |  | *SD* | 8.25 | 7.07 | 5.40 |  |
| RT (ms; Go block) | 22 | min. | 282.36 | 345.07 | 347.48 | *F*_(2, 42)_ = 1.07, *p =* 0.35, ƞ^2^ = 0.05 |
|  |  | max. | 611.39 | 673.80 | 600.96 |  |
|  |  | *M* | 451.20 | 477.25 | 473.70 |  |
|  |  | *SD* | 87.27 | 95.43 | 71.86 |  |
| RT (ms; Go/No-go block) | 22 | min. | 420.80 | 461.31 | 481.55 | *F*_(2, 42)_ = 0.01, *p =* 0.99, ƞ^2^ = 0.001 |
|  |  | max. | 658.11 | 661.33 | 652.14 |  |
|  |  | *M* | 563.05 | 564.37 | 564.97 |  |
|  |  | *SD* | 68.60 | 59.45 | 44.61 |  |

*Note.* M = mean; SD = standard deviation; FA = false alarm; RT = response time

**Table S2** Summary of analyses of incentive Go/No-go task performance by condition and gender

| **Variable** | **Gender** | ***n*** |  | **Control** | **Social** | **Nonsocial** | **ANOVA** | | |
| --- | --- | --- | --- | --- | --- | --- | --- | --- | --- |
|  |  |  |  |  |  |  | **Within-subjects effects** | **Between-subject factors** | **Interaction** |
| Hits (%; Go block) | Boy | 12 | *M* | 80.00 | 78.33 | 82.41 | *F*_(2, 44)_ = 0.13,  *p =* 0.88,  ƞ^2^ = 0.006 | *F*_(1, 22)_ = 0.97,  *p =* 0.34,  ƞ^2^ = 0.04 | *F*_(2, 44)_ = 0.43,  *p =* 0.66,  ƞ^2^ = 0.02 |
|  |  |  | *SD* | 10.76 | 12.40 | 18.43 |  |  |  |
|  | Girl | 12 | *M* | 84.07 | 85.93 | 84.45 |  |  |  |
|  |  |  | *SD* | 13.50 | 12.29 | 16.68 |  |  |  |
|  | Total | 24 | *M* | 82.04 | 82.13 | 83.43 |  |  |  |
|  |  |  | *SD* | 12.12 | 12.68 | 17.23 |  |  |  |
| Hits (%; Go/No-go block) | Boy | 12 | *M* | 75.31 | 79.32 | 76.54 | *F*_(2, 44)_ = 0.27,  *p =* 0.77,  ƞ^2^ = 0.01 | *F*_(1, 22)_ = 0.40,  *p =* 0.54,  ƞ^2^ = 0.02 | *F*_(2, 44)_ = 0.31,  *p =* 0.73,  ƞ^2^ = 0.01 |
|  |  |  | *SD* | 14.24 | 13.44 | 12.67 |  |  |  |
|  | Girl | 12 | *M* | 71.92 | 72.22 | 74.08 |  |  |  |
|  |  |  | *SD* | 22.97 | 22.80 | 23.74 |  |  |  |
|  | Total | 24 | *M* | 73.61 | 75.77 | 75.31 |  |  |  |
|  |  |  | *SD* | 18.77 | 18.66 | 18.65 |  |  |  |
| FA (%; Go/No-go block) | Boy | 12 | *M* | 6.95 | 6.48 | 5.56 | *F*_(2, 44)_ = 0.21,  *p =* 0.81,  ƞ^2^ = 0.01 | *F*_(1, 22)_ = 0.22,  *p =* 0.88,  ƞ^2^ = 0.001 | *F*_(2, 44)_ = 0.01,  *p =* 0.99,  ƞ^2^ = 0.001 |
|  |  |  | *SD* | 9.21 | 8.15 | 5.30 |  |  |  |
|  | Girl | 12 | *M* | 6.48 | 6.02 | 5.56 |  |  |  |
|  |  |  | *SD* | 7.04 | 6.47 | 6.27 |  |  |  |
|  | Total | 24 | *M* | 6.71 | 6.25 | 5.56 |  |  |  |
|  |  |  | *SD* | 8.02 | 7.20 | 5.67 |  |  |  |
| RT (ms; Go block) | Boy | 12 | *M* | 420.11 | 453.07 | 454.29 | *F*_(2, 44)_ = 1.22,  *p =* 0.31,  ƞ^2^ = 0.05 | *F*_(1, 22)_ = 1.74,  *p =* 0.20,  ƞ^2^ = 0.07 | *F*_(2, 44)_ = 0.38,  *p =* 0.68,  ƞ^2^ = 0.02 |
|  |  |  | *SD* | 87.21 | 100.42 | 80.40 |  |  |  |
|  | Girl | 12 | *M* | 469.90 | 494.03 | 472.58 |  |  |  |
|  |  |  | *SD* | 81.84 | 82.48 | 78.32 |  |  |  |
|  | Total | 24 | *M* | 445.00 | 473.55 | 463.44 |  |  |  |
|  |  |  | *SD* | 86.53 | 92.27 | 78.18 |  |  |  |
| RT (ms; Go/No-go block) | Boy | 12 | *M* | 555.16 | 569.32 | 565.47 | *F*_(2, 44)_ = 0.004,  *p =* 1.00,  ƞ^2^ = 0.00 | *F*_(1, 22)_ = 0.19,  *p =* 0.67,  ƞ^2^ = 0.01 | *F*_(2, 44)_ = 0.69,  *p =* 0.51,  ƞ^2^ = 0.03 |
|  |  |  | *SD* | 59.56 | 48.83 | 41.80 |  |  |  |
|  | Girl | 12 | *M* | 562.36 | 549.34 | 551.03 |  |  |  |
|  |  |  | *SD* | 78.19 | 73.94 | 58.31 |  |  |  |
|  | Total | 24 | *M* | 558.76 | 559.33 | 558.25 |  |  |  |
|  |  |  | *SD* | 68.08 | 62.12 | 50.16 |  |  |  |

*Note.* M = mean; SD = standard deviation; FA = false alarm; RT = response time

**Table S3** Summary of analyses of the difference in oxy-Hb responses between the Go/No-go block and the Go block

| **Channel** | ***n*** |  | **Control** | **Social** | **Nonsocial** | **Statistics** |
| --- | --- | --- | --- | --- | --- | --- |
| 3 | 25 | min. | -0.0042 | -0.0028 | -0.0027 | *F*_(2, 48)_ = 6.82, *p =* 0.002, ƞ^2^ = 0.22 |
|  |  | max. | 0.0034 | 0.0066 | 0.0037 |  |
|  |  | *M* | -0.0002 | 0.0018 | 0.0008 |  |
|  |  | *SD* | 0.0019 | 0.0022 | 0.0017 |  |
| 4 | 24 | min. | -0.0045 | -0.0037 | -0.0044 | *F*_(1.54, 35.34)_ = 1.05, *p =* 0.34, ƞ^2^ = 0.04 |
|  |  | max. | 0.0068 | 0.0071 | 0.0055 |  |
|  |  | *M* | 0.0003 | 0.0013 | 0.0006 |  |
|  |  | *SD* | 0.0024 | 0.0030 | 0.0029 |  |
| 6 | 25 | min. | -0.0057 | -0.0030 | -0.0050 | *F*_(2, 48)_ = 4.01, *p =* 0.03, ƞ^2^ = 0.14 |
|  |  | max. | 0.0032 | 0.0052 | 0.0061 |  |
|  |  | *M* | -0.0005 | 0.0011 | 0.0002 |  |
|  |  | *SD* | 0.0024 | 0.0020 | 0.0029 |  |
| 7 | 23 | min. | -0.0043 | -0.0049 | -0.0052 | *F*_(2, 44)_ = 3.45, *p =* 0.04, ƞ^2^ = 0.14 |
|  |  | max. | 0.0043 | 0.0053 | 0.0034 |  |
|  |  | *M* | -0.0002 | 0.0010 | -0.0005 |  |
|  |  | *SD* | 0.0019 | 0.0023 | 0.0022 |  |
| 9 | 25 | min. | -0.0041 | -0.0037 | -0.0028 | *F*_(2, 48)_ = 0.59, *p =* 0.56, ƞ^2^ = 0.02 |
|  |  | max. | 0.0041 | 0.0067 | 0.0054 |  |
|  |  | *M* | 0.0001 | 0.0008 | 0.0006 |  |
|  |  | *SD* | 0.0020 | 0.0023 | 0.0021 |  |
| 10 | 24 | min. | -0.0053 | -0.0040 | -0.0039 | *F*_(2, 46)_ = 3.82, *p =* 0.03, ƞ^2^ = 0.14 |
|  |  | max. | 0.0044 | 0.0066 | 0.0051 |  |
|  |  | *M* | 0.0002 | 0.0018 | 0.0007 |  |
|  |  | *SD* | 0.0021 | 0.0024 | 0.0022 |  |

*Note.* M = mean; SD = standard deviation

**Table S4** Summary of analyses of the difference in deoxy-Hb responses between the Go/No-go block and the Go block

| **Channel** | ***n*** |  | **Control** | **Social** | **Nonsocial** | **Statistics** |
| --- | --- | --- | --- | --- | --- | --- |
| 3 | 25 | min. | -0.0020 | -0.0039 | -0.0022 | *F*_(2, 48)_ = 6.82, *p =* 0.002, ƞ^2^ = 0.22 |
|  |  | max. | 0.0025 | 0.0017 | 0.0016 |  |
|  |  | *M* | 0.0001 | -0.0011 | -0.0005 |  |
|  |  | *SD* | 0.0011 | 0.0013 | 0.0010 |  |
| 4 | 24 | min. | -0.0041 | -0.0043 | -0.0033 | *F*_(1.54, 35.34)_ = 1.05, *p =* 0.34, ƞ^2^ = 0.04 |
|  |  | max. | 0.0027 | 0.0022 | 0.0026 |  |
|  |  | *M* | -0.0002 | -0.0008 | -0.0003 |  |
|  |  | *SD* | 0.0014 | 0.0018 | 0.0018 |  |
| 6 | 25 | min. | -0.0019 | -0.0031 | -0.0036 | *F*_(2, 48)_ = 4.01, *p =* 0.03, ƞ^2^ = 0.14 |
|  |  | max. | 0.0034 | 0.0018 | 0.0030 |  |
|  |  | *M* | 0.0003 | -0.0007 | -0.0001 |  |
|  |  | *SD* | 0.0014 | 0.0012 | 0.0018 |  |
| 7 | 23 | min. | -0.0026 | -0.0032 | -0.0020 | *F*_(2, 44)_ = 3.45, *p =* 0.04, ƞ^2^ = 0.14 |
|  |  | max. | 0.0026 | 0.0030 | 0.0031 |  |
|  |  | *M* | 0.0001 | -0.0006 | 0.0003 |  |
|  |  | *SD* | 0.0012 | 0.0014 | 0.0013 |  |
| 9 | 25 | min. | -0.0025 | -0.0040 | -0.0032 | *F*_(2, 48)_ = 0.59, *p =* 0.56, ƞ^2^ = 0.02 |
|  |  | max. | 0.0025 | 0.0022 | 0.0017 |  |
|  |  | *M* | -0.0001 | -0.0005 | -0.0003 |  |
|  |  | *SD* | 0.0012 | 0.0014 | 0.0013 |  |
| 10 | 24 | min. | -0.0027 | -0.0040 | -0.0031 | *F*_(2, 46)_ = 3.82, *p =* 0.03, ƞ^2^ = 0.14 |
|  |  | max. | 0.0032 | 0.0024 | 0.0024 |  |
|  |  | *M* | -0.0001 | -0.0011 | -0.0004 |  |
|  |  | *SD* | 0.0013 | 0.0014 | 0.0013 |  |

*Note.* M = mean; SD = standard deviation

**Table S5** Summary of analyses of oxy-Hb changes by condition when fathers’ faces were excluding

| **Channel** | ***n*** |  | **Control** | | **Social** | | **Nonsocial** | |  | **ANOVA** | | |
| --- | --- | --- | --- | --- | --- | --- | --- | --- | --- | --- | --- | --- |
|  |  |  | **Go** | **Go/No-go** | **Go** | **Go/No-go** | **Go** | **Go/No-go** |  | **Condition** | **Block** | **Interaction** |
| 3 | 23 | min. | -0.0042 | -0.0029 | -0.0042 | -0.0035 | -0.0055 | -0.0038 |  | *F*_(2, 44)_ = 0.26,  *p =* 0.77,  ƞ^2^ = 0.01 | *F*_(1, 22)_ = 17.11,  *p <* 0.001,  ƞ^2^ = 0.44 | *F*_(2, 44)_ = 5.91,  *p =* 0.005,  ƞ^2^ = 0.21 |
|  |  | max. | 0.0042 | 0.0030 | 0.0024 | 0.0057 | 0.0040 | 0.0035 |  |  |  |  |
|  |  | *M* | 0.0001 | 0.0001 | -0.0007 | 0.0013 | -0.0004 | 0.0004 |  |  |  |  |
|  |  | *SD* | 0.0018 | 0.0016 | 0.0015 | 0.0024 | 0.0018 | 0.0019 |  |  |  |  |
| 4 | 23 | min. | -0.0036 | -0.0109 | -0.0051 | -0.0038 | -0.0038 | -0.0030 |  | *F*_(2, 44)_ = 0.75,  *p =* 0.48,  ƞ^2^ = 0.03 | *F*_(1, 22)_ = 1.13,  *p =* 0.30,  ƞ^2^ = 0.05 | *F*_(2, 44)_ = 0.96,  *p =* 0.39,  ƞ^2^ = 0.04 |
|  |  | max. | 0.0051 | 0.0054 | 0.0024 | 0.0064 | 0.0035 | 0.0134 |  |  |  |  |
|  |  | *M* | 0.0003 | 0.0001 | 0.0002 | 0.0013 | 0.0002 | 0.0008 |  |  |  |  |
|  |  | *SD* | 0.0021 | 0.0034 | 0.0016 | 0.0026 | 0.0016 | 0.0029 |  |  |  |  |
| 6 | 23 | min. | -0.0038 | -0.0056 | -0.0030 | -0.0024 | -0.0030 | -0.0029 |  | *F*_(1.56, 34.22)_ = 0.94,  *p =* 0.40,  ƞ^2^ = 0.04 | *F*_(1,22)_ = 1.06,  *p =* 0.32,  ƞ^2^ = 0.05 | *F*_(2, 44)_ = 2.80,  *p =* 0.07,  ƞ^2^ = 0.11 |
|  |  | max. | 0.0052 | 0.0043 | 0.0027 | 0.0043 | 0.0134 | 0.0041 |  |  |  |  |
|  |  | *M* | 0.0002 | -0.0001 | 0.0001 | 0.0012 | 0.0006 | 0.0009 |  |  |  |  |
|  |  | *SD* | 0.0017 | 0.0022 | 0.0013 | 0.0019 | 0.0032 | 0.0031 |  |  |  |  |
| 7 | 21 | min. | -0.0017 | -0.0026 | -0.0046 | -0.0027 | -0.0029 | -0.0064 |  | *F*_(2, 40)_ = 0.73,  *p =* 0.49,  ƞ^2^ = 0.04 | *F*_(1, 20)_ = 0.17,  *p =* 0.69,  ƞ^2^ = 0.008 | *F*_(2, 40)_ = 2.00,  *p =* 0.15,  ƞ^2^ = 0.09 |
|  |  | max. | 0.0048 | 0.0039 | 0.0025 | 0.0022 | 0.0041 | 0.0016 |  |  |  |  |
|  |  | *M* | 0.0003 | 0.0001 | 0.0001 | 0.0009 | 0.0002 | -0.0002 |  |  |  |  |
|  |  | *SD* | 0.0014 | 0.0017 | 0.0017 | 0.0019 | 0.0016 | 0.0022 |  |  |  |  |
| 9 | 23 | min. | -0.0028 | -0.0046 | -0.0013 | -0.0023 | -0.0064 | -0.0030 |  | *F*_(2, 44)_ = 3.11,  *p =* 0.05,  ƞ^2^ = 0.12 | *F*_(1, 22)_ = 4.28,  *p =* 0.05,  ƞ^2^ = 0.16 | *F*_(2, 44)_ = 0.56,  *p =* 0.58,  ƞ^2^ = 0.03 |
|  |  | max. | 0.0010 | 0.0025 | 0.0112 | 0.0075 | 0.0016 | 0.0020 |  |  |  |  |
|  |  | *M* | -0.0004 | -0.0002 | 0.0004 | 0.0011 | -0.0007 | 0.0000 |  |  |  |  |
|  |  | *SD* | 0.0009 | 0.0016 | 0.0026 | 0.0024 | 0.0016 | 0.0027 |  |  |  |  |
| 10 | 23 | min. | -0.0031 | -0.0027 | -0.0033 | -0.0033 | -0.0030 | -0.0035 |  | *F*_(2, 44)_ = 1.63,  *p =* 0.21,  ƞ^2^ = 0.07 | *F*_(1, 22)_ = 8.23,  *p =* 0.01,  ƞ^2^ = 0.27 | *F*_(2, 46)_ = 3.34,  *p =* 0.05,  ƞ^2^ = 0.13 |
|  |  | max. | 0.0028 | 0.0022 | 0.0017 | 0.0058 | 0.0020 | 0.0040 |  |  |  |  |
|  |  | *M* | 0.0000 | 0.0001 | -0.0004 | 0.0013 | -0.0005 | 0.0003 |  |  |  |  |
|  |  | *SD* | 0.0012 | 0.0014 | 0.0012 | 0.0021 | 0.0012 | 0.0021 |  |  |  |  |

*Note.* M = mean; SD = standard deviation

**Table S6** Summary of analyses of deoxy-Hb changes by condition when fathers’ faces were excluding

| **Channel** | ***n*** |  | **Control** | | **Social** | | **Nonsocial** | |  | **ANOVA** | | |
| --- | --- | --- | --- | --- | --- | --- | --- | --- | --- | --- | --- | --- |
|  |  |  | **Go** | **Go/No-go** | **Go** | **Go/No-go** | **Go** | **Go/No-go** |  | **Condition** | **Block** | **Interaction** |
| 3 | 23 | min. | -0.0025 | -0.0018 | -0.0014 | -0.0034 | -0.0024 | -0.0024 |  | *F*_(2, 44)_ = 0.26,  *p =* 0.77,  ƞ^2^ = 0.01 | *F*_(1, 22)_ = 17.11,  *p <* 0.001,  ƞ^2^ = 0.44 | *F*_(2, 44)_ = 5.91,  *p =* 0.005,  ƞ^2^ = 0.21 |
|  |  | max. | 0.0025 | 0.0018 | 0.0025 | 0.0021 | 0.0033 | 0.0021 |  |  |  |  |
|  |  | *M* | -0.0001 | -0.0001 | 0.0004 | -0.0008 | 0.0002 | -0.0003 |  |  |  |  |
|  |  | *SD* | 0.0011 | 0.0010 | 0.0009 | 0.0015 | 0.0011 | 0.0012 |  |  |  |  |
| 4 | 23 | min. | -0.0030 | -0.0032 | -0.0014 | -0.0038 | -0.0021 | -0.0044 |  | *F*_(2, 44)_ = 0.75,  *p =* 0.48,  ƞ^2^ = 0.03 | *F*_(1, 22)_ = 1.13,  *p =* 0.30,  ƞ^2^ = 0.05 | *F*_(2, 44)_ = 0.96,  *p =* 0.39,  ƞ^2^ = 0.04 |
|  |  | max. | 0.0022 | 0.0065 | 0.0030 | 0.0023 | 0.0023 | 0.0020 |  |  |  |  |
|  |  | *M* | -0.0002 | -0.0001 | -0.0001 | -0.0008 | -0.0001 | -0.0005 |  |  |  |  |
|  |  | *SD* | 0.0012 | 0.0020 | 0.0010 | 0.0016 | 0.0010 | 0.0018 |  |  |  |  |
| 6 | 23 | min. | -0.0031 | -0.0026 | -0.0016 | -0.0026 | -0.0080 | -0.0050 |  | *F*_(1.56, 34.22)_ = 0.94,  *p =* 0.40,  ƞ^2^ = 0.04 | *F*_(1,22)_ = 1.06,  *p =* 0.32,  ƞ^2^ = 0.05 | *F*_(2, 44)_ = 2.80,  *p =* 0.07,  ƞ^2^ = 0.11 |
|  |  | max. | 0.0023 | 0.0034 | 0.0018 | 0.0014 | 0.0018 | 0.0035 |  |  |  |  |
|  |  | *M* | -0.0001 | 0.0000 | -0.0001 | -0.0007 | -0.0003 | -0.0005 |  |  |  |  |
|  |  | *SD* | 0.0010 | 0.0013 | 0.0008 | 0.0011 | 0.0019 | 0.0019 |  |  |  |  |
| 7 | 21 | min. | -0.0029 | -0.0024 | -0.0027 | -0.0030 | -0.0024 | -0.0023 |  | *F*_(2, 40)_ = 0.73,  *p =* 0.49,  ƞ^2^ = 0.04 | *F*_(1, 20)_ = 0.17,  *p =* 0.69,  ƞ^2^ = 0.008 | *F*_(2, 40)_ = 2.00,  *p =* 0.15,  ƞ^2^ = 0.09 |
|  |  | max. | 0.0010 | 0.0016 | 0.0015 | 0.0016 | 0.0017 | 0.0026 |  |  |  |  |
|  |  | *M* | -0.0002 | -0.0001 | -0.0001 | -0.0006 | -0.0001 | 0.0001 |  |  |  |  |
|  |  | *SD* | 0.0008 | 0.0010 | 0.0010 | 0.0012 | 0.0009 | 0.0013 |  |  |  |  |
| 9 | 23 | min. | -0.0006 | -0.0015 | -0.0067 | -0.0045 | -0.0010 | -0.0029 |  | *F*_(2, 44)_ = 3.11,  *p =* 0.05,  ƞ^2^ = 0.12 | *F*_(1, 22)_ = 4.28,  *p =* 0.05,  ƞ^2^ = 0.16 | *F*_(2, 44)_ = 0.56,  *p =* 0.58,  ƞ^2^ = 0.03 |
|  |  | max. | 0.0017 | 0.0028 | 0.0008 | 0.0014 | 0.0038 | 0.0050 |  |  |  |  |
|  |  | *M* | 0.0002 | 0.0001 | -0.0002 | -0.0007 | 0.0004 | 0.0000 |  |  |  |  |
|  |  | *SD* | 0.0005 | 0.0010 | 0.0015 | 0.0014 | 0.0009 | 0.0016 |  |  |  |  |
| 10 | 23 | min. | -0.0017 | -0.0013 | -0.0010 | -0.0035 | -0.0012 | -0.0022 |  | *F*_(2, 44)_ = 1.63,  *p =* 0.21,  ƞ^2^ = 0.07 | *F*_(1, 22)_ = 8.23,  *p =* 0.01,  ƞ^2^ = 0.27 | *F*_(2, 46)_ = 3.34,  *p =* 0.05,  ƞ^2^ = 0.13 |
|  |  | max. | 0.0019 | 0.0016 | 0.0020 | 0.0020 | 0.0018 | 0.0025 |  |  |  |  |
|  |  | *M* | 0.0000 | 0.0000 | 0.0002 | -0.0008 | 0.0003 | -0.0002 |  |  |  |  |
|  |  | *SD* | 0.0007 | 0.0008 | 0.0007 | 0.0013 | 0.0007 | 0.0012 |  |  |  |  |

*Note.* M = mean; SD = standard deviation

**Table S7** Summary of analyses of oxy-Hb changes by condition and gender

| **Ch** | **Gender** | ***n*** |  | **Control** | | **Social** | | **Nonsocial** | | **ANOVA** | |
| --- | --- | --- | --- | --- | --- | --- | --- | --- | --- | --- | --- |
|  |  |  |  | **Go** | **Go/**  **No-go** | **Go** | **Go/**  **No-go** | **Go** | **Go/**  **No-go** |  |  |
| 3 | Boy | 12 | *M* | 0.0004 | 0.0005 | -0.0004 | 0.0011 | -0.0001 | 0.0014 | Condition | *F*_(2, 46)_ = 0.26, *p =* 0.77, ƞ^2^ = 0.01 |
|  |  |  | *SD* | 0.0017 | 0.0014 | 0.0015 | 0.0029 | 0.0009 | 0.0013 | Block | *F*_(1, 23)_ = 11.92, *p =* 0.002, ƞ^2^ = 0.34 |
|  | Girl | 13 | *M* | -0.0002 | -0.0005 | -0.0010 | 0.0011 | -0.0008 | -0.0007 | Gender | *F*_(1, 23)_ = 3.23, *p =* 0.09, ƞ^2^ = 0.12 |
|  |  |  | *SD* | 0.0017 | 0.0019 | 0.0015 | 0.0020 | 0.0023 | 0.0022 | Condition*Block | *F*_(2, 46)_ = 6.87, *p =* 0.002, ƞ^2^ = 0.23 |
|  | Total | 25 | *M* | 0.0001 | -0.0001 | -0.0007 | 0.0011 | -0.0005 | 0.0003 | Condition*Gender | *F*_(2, 46)_ = 0.95, *p =* 0.39, ƞ^2^ = 0.04 |
|  |  |  | *SD* | 0.0017 | 0.0017 | 0.0015 | 0.0024 | 0.0017 | 0.0021 | Block*Gender | *F*_(1, 23)_ = 0.66, *p =* 0.43, ƞ^2^ = 0.03 |
|  |  |  |  |  |  |  |  |  |  | Condition*Block*Gender | *F*_(2, 46)_ = 1.75, *p =* 0.19, ƞ^2^ = 0.07 |
| 4 | Boy | 12 | *M* | 0.0010 | 0.0005 | 0.0003 | 0.0024 | 0.0005 | 0.0018 | Condition | *F*_(2, 46)_ = 1.03, *p =* 0.37, ƞ^2^ = 0.04 |
|  |  |  | *SD* | 0.0023 | 0.0043 | 0.0012 | 0.0025 | 0.0011 | 0.0025 | Block | *F*_(1, 23)_ = 1.36, *p =* 0.26, ƞ^2^ = 0.56 |
|  | Girl | 13 | *M* | -0.0003 | -0.0004 | -0.0001 | 0.0004 | -0.0001 | -0.0002 | Gender | *F*_(1, 23)_ = 7.01, *p =* 0.01, ƞ^2^ = 0.23 |
|  |  |  | *SD* | 0.0015 | 0.0020 | 0.0019 | 0.0023 | 0.0019 | 0.0030 | Condition*Block | *F*_(2, 46)_ = 1.68, *p =* 0.20, ƞ^2^ = 0.07 |
|  | Total | 25 | *M* | 0.0003 | 0.0000 | 0.0001 | 0.0013 | 0.0002 | 0.0008 | Condition*Gender | *F*_(2, 46)_ = 0.03, *p =* 0.97, ƞ^2^ = 0.001 |
|  |  |  | *SD* | 0.0020 | 0.0033 | 0.0016 | 0.0026 | 0.0016 | 0.0029 | Block*Gender | *F*_(1, 23)_ = 0.92, *p =* 0.35, ƞ^2^ = 0.04 |
|  |  |  |  |  |  |  |  |  |  | Condition*Block*Gender | *F*_(2, 46)_ = 0.75, *p =* 0.48, ƞ^2^ = 0.03 |
| 6 | Boy | 12 | *M* | 0.0008 | 0.0005 | 0.0001 | 0.0016 | 0.0014 | 0.0020 | Condition | *F*_(2, 46)_ = 0.93, *p =* 0.40, ƞ^2^ = 0.40 |
|  |  |  | *SD* | 0.0018 | 0.0022 | 0.0010 | 0.0019 | 0.0040 | 0.0031 | Block | *F*_(1, 23)_ = 0.62, *p =* 0.44, ƞ^2^ = 0.03 |
|  | Girl | 13 | *M* | -0.0002 | -0.0008 | 0.0000 | 0.0008 | -0.0002 | -0.0004 | Gender | *F*_(1, 23)_ = 6.36, *p =* 0.02, ƞ^2^ = 0.22 |
|  |  |  | *SD* | 0.0014 | 0.0020 | 0.0015 | 0.0018 | 0.0017 | 0.0028 | Condition*Block | *F*_(2, 46)_ = 3.85, *p =* 0.03, ƞ^2^ = 0.14 |
|  | Total | 25 | *M* | 0.0003 | -0.0002 | 0.0000 | 0.0012 | 0.0005 | 0.0007 | Condition*Gender | *F*_(2, 46)_ = 1.19, *p =* 0.31, ƞ^2^ = 0.50 |
|  |  |  | *SD* | 0.0016 | 0.0022 | 0.0013 | 0.0019 | 0.0031 | 0.0031 | Block*Gender | *F*_(1, 23)_ = 0.55, *p =* 0.57, ƞ^2^ = 0.03 |
|  |  |  |  |  |  |  |  |  |  | Condition*Block*Gender | *F*_(2, 46)_ = 0.03, *p =* 0.97, ƞ^2^ = 0.001 |
| 7 | Boy | 11 | *M* | 0.0006 | 0.0004 | -0.0001 | 0.0019 | 0.0005 | 0.0001 | Condition | *F*_(2, 42)_ = 0.57, *p =* 0.77, ƞ^2^ = 0.03 |
|  |  |  | *SD* | 0.0017 | 0.0021 | 0.0014 | 0.0019 | 0.0008 | 0.0022 | Block | *F*_(1, 21)_ = 0.12, *p =* 0.73, ƞ^2^ = 0.01 |
|  | Girl | 12 | *M* | 0.0002 | -0.0001 | 0.0000 | 0.0000 | 0.0000 | -0.0005 | Gender | *F*_(1, 21)_ = 3.21, *p =* 0.09, ƞ^2^ = 0.13 |
|  |  |  | *SD* | 0.0011 | 0.0012 | 0.0020 | 0.0015 | 0.0020 | 0.0024 | Condition*Block | *F*_(2, 42)_ = 3.78, *p =* 0.03, ƞ^2^ = 0.15 |
|  | Total | 23 | *M* | 0.0004 | 0.0001 | 0.0000 | 0.0009 | 0.0002 | -0.0002 | Condition*Gender | *F*_(2, 42)_ = 0.16, *p =* 0.86, ƞ^2^ = 0.01 |
|  |  |  | *SD* | 0.0014 | 0.0017 | 0.0017 | 0.0019 | 0.0015 | 0.0023 | Block*Gender | *F*_(1, 21)_ = 1.59, *p =* 0.22, ƞ^2^ = 0.07 |
|  |  |  |  |  |  |  |  |  |  | Condition*Block*Gender | *F*_(2, 42)_ = 1.76, *p =* 0.19, ƞ^2^ = 0.08 |
| 9 | Boy | 12 | *M* | -0.0002 | 0.0002 | 0.0007 | 0.0022 | -0.0003 | 0.0007 | Condition | *F*_(2, 44)_ = 2.85, *p =* 0.07, ƞ^2^ = 0.012 |
|  |  |  | *SD* | 0.0010 | 0.0018 | 0.0033 | 0.0027 | 0.0014 | 0.0021 | Block | *F*_(1, 22)_ = 5.32, *p =* 0.03, ƞ^2^ = 0.20 |
|  | Girl | 12 | *M* | -0.0005 | -0.0003 | 0.0000 | 0.0001 | -0.0005 | -0.0001 | Gender | *F*_(1, 22)_ = 3.56, *p =* 0.07, ƞ^2^ = 0.14 |
|  |  |  | *SD* | 0.0007 | 0.0019 | 0.0013 | 0.0014 | 0.0011 | 0.0020 | Condition*Block | *F*_(2, 44)_ = 0.42, *p =* 0.66, ƞ^2^ = 0.02 |
|  | Total | 24 | *M* | -0.0003 | 0.0000 | 0.0003 | 0.0011 | -0.0004 | 0.0003 | Condition*Gender | *F*_(2, 44)_ = 0.98, *p =* 0.38, ƞ^2^ = 0.04 |
|  |  |  | *SD* | 0.0009 | 0.0019 | 0.0025 | 0.0024 | 0.0012 | 0.0020 | Block*Gender | *F*_(1, 22)_ = 1.80, *p =* 0.19, ƞ^2^ = 0.08 |
|  |  |  |  |  |  |  |  |  |  | Condition*Block*Gender | *F*_(2, 44)_ = 0.56, *p =* 0.57, ƞ^2^ = 0.03 |
| 10 | Boy | 12 | *M* | 0.0003 | 0.0006 | -0.0008 | 0.0020 | -0.0003 | 0.0007 | Condition | *F*_(2, 44)_ = 1.58, *p =* 0.22, ƞ^2^ = 0.07 |
|  |  |  | *SD* | 0.0013 | 0.0014 | 0.0010 | 0.0018 | 0.0014 | 0.0021 | Block | *F*_(1, 22)_ = 10.72, *p =* 0.003, ƞ^2^ = 0.33 |
|  | Girl | 12 | *M* | -0.0003 | -0.0004 | -0.0001 | 0.0008 | -0.0005 | -0.0001 | Gender | *F*_(1, 22)_ = 3.97, *p =* 0.06, ƞ^2^ = 0.15 |
|  |  |  | *SD* | 0.0010 | 0.0012 | 0.0013 | 0.0023 | 0.0011 | 0.0020 | Condition*Block | *F*_(2, 44)_ = 3.77, *p =* 0.03, ƞ^2^ = 0.15 |
|  | Total | 24 | *M* | 0.0000 | 0.0001 | -0.0004 | 0.0014 | -0.0004 | 0.0003 | Condition*Gender | *F*_(2, 44)_ = 0.34, *p =* 0.72, ƞ^2^ = 0.02 |
|  |  |  | *SD* | 0.0012 | 0.0014 | 0.0012 | 0.0021 | 0.0012 | 0.0020 | Block*Gender | *F*_(1, 22)_ = 2.77, *p =* 0.11, ƞ^2^ = 0.11 |
|  |  |  |  |  |  |  |  |  |  | Condition*Block*Gender | *F*_(2, 44)_ = 0.74, *p =* 0.48, ƞ^2^ = 0.03 |

*Note.* Ch = Channel; M = mean; SD = standard deviation

**Table S8** Summary of Analyses of deoxy-Hb changes by condition and gender

| **Ch** | **Gender** | ***n*** |  | **Control** | | **Social** | | **Nonsocial** | | **ANOVA** | |
| --- | --- | --- | --- | --- | --- | --- | --- | --- | --- | --- | --- |
|  |  |  |  | **Go** | **Go/**  **No-go** | **Go** | **Go/**  **No-go** | **Go** | **Go/**  **No-go** |  |  |
| 3 | Boy | 12 | *M* | -0.0002 | -0.0003 | 0.0002 | -0.0007 | 0.0001 | -0.0008 | Condition | *F*_(2, 46)_ = 0.26, *p =* 0.77, ƞ^2^ = 0.01 |
|  |  |  | *SD* | 0.0010 | 0.0008 | 0.0009 | 0.0017 | 0.0005 | 0.0008 | Block | *F*_(1, 23)_ = 11.92, *p =* 0.002, ƞ^2^ = 0.34 |
|  | Girl | 13 | *M* | 0.0001 | 0.0003 | 0.0006 | -0.0007 | 0.0005 | 0.0004 | Gender | *F*_(1, 23)_ = 3.23, *p =* 0.09, ƞ^2^ = 0.12 |
|  |  |  | *SD* | 0.0010 | 0.0011 | 0.0009 | 0.0012 | 0.0014 | 0.0013 | Condition*Block | *F*_(2, 46)_ = 6.87, *p =* 0.002, ƞ^2^ = 0.23 |
|  | Total | 25 | *M* | -0.0001 | 0.0000 | 0.0004 | -0.0007 | 0.0003 | -0.0002 | Condition*Gender | *F*_(2, 46)_ = 0.95, *p =* 0.39, ƞ^2^ = 0.04 |
|  |  |  | *SD* | 0.0010 | 0.0010 | 0.0009 | 0.0015 | 0.0010 | 0.0013 | Block*Gender | *F*_(1, 23)_ = 0.66, *p =* 0.43, ƞ^2^ = 0.03 |
|  |  |  |  |  |  |  |  |  |  | Condition*Block*Gender | *F*_(2, 46)_ = 1.75, *p =* 0.19, ƞ^2^ = 0.07 |
| 4 | Boy | 12 | *M* | -0.0006 | -0.0003 | -0.0002 | -0.0014 | -0.0003 | -0.0011 | Condition | *F*_(2, 46)_ = 1.03, *p =* 0.37, ƞ^2^ = 0.04 |
|  |  |  | *SD* | 0.0014 | 0.0026 | 0.0007 | 0.0015 | 0.0006 | 0.0015 | Block | *F*_(1, 23)_ = 1.36, *p =* 0.26, ƞ^2^ = 0.56 |
|  | Girl | 13 | *M* | 0.0002 | 0.0003 | 0.0001 | -0.0002 | 0.0000 | 0.0001 | Gender | *F*_(1, 23)_ = 7.01, *p =* 0.01, ƞ^2^ = 0.23 |
|  |  |  | *SD* | 0.0009 | 0.0012 | 0.0012 | 0.0014 | 0.0012 | 0.0018 | Condition*Block | *F*_(2, 46)_ = 1.68, *p =* 0.20, ƞ^2^ = 0.07 |
|  | Total | 25 | *M* | -0.0002 | 0.0000 | -0.0001 | -0.0008 | -0.0001 | -0.0005 | Condition*Gender | *F*_(2, 46)_ = 0.03, *p =* 0.97, ƞ^2^ = 0.001 |
|  |  |  | *SD* | 0.0012 | 0.0020 | 0.0010 | 0.0015 | 0.0009 | 0.0017 | Block*Gender | *F*_(1, 23)_ = 0.92, *p =* 0.35, ƞ^2^ = 0.04 |
|  |  |  |  |  |  |  |  |  |  | Condition*Block*Gender | *F*_(2, 46)_ = 0.75, *p =* 0.48, ƞ^2^ = 0.03 |
| 6 | Boy | 12 | *M* | -0.0005 | -0.0003 | -0.0001 | -0.0009 | -0.0008 | -0.0012 | Condition | *F*_(2, 46)_ = 0.93, *p =* 0.40, ƞ^2^ = 0.40 |
|  |  |  | *SD* | 0.0011 | 0.0013 | 0.0006 | 0.0012 | 0.0024 | 0.0019 | Block | *F*_(1, 23)_ = 0.62, *p =* 0.44, ƞ^2^ = 0.03 |
|  | Girl | 13 | *M* | 0.0001 | 0.0005 | 0.0000 | -0.0005 | 0.0001 | 0.0002 | Gender | *F*_(1, 23)_ = 6.36, *p =* 0.02, ƞ^2^ = 0.22 |
|  |  |  | *SD* | 0.0008 | 0.0012 | 0.0009 | 0.0011 | 0.0010 | 0.0017 | Condition*Block | *F*_(2, 46)_ = 3.85, *p =* 0.03, ƞ^2^ = 0.14 |
|  | Total | 25 | *M* | -0.0002 | 0.0001 | 0.0000 | -0.0007 | -0.0003 | -0.0004 | Condition*Gender | *F*_(2, 46)_ = 1.19, *p =* 0.31, ƞ^2^ = 0.50 |
|  |  |  | *SD* | 0.0010 | 0.0013 | 0.0008 | 0.0011 | 0.0018 | 0.0019 | Block*Gender | *F*_(1, 23)_ = 0.55, *p =* 0.57, ƞ^2^ = 0.03 |
|  |  |  |  |  |  |  |  |  |  | Condition*Block*Gender | *F*_(2, 46)_ = 0.03, *p =* 0.97, ƞ^2^ = 0.001 |
| 7 | Boy | 11 | *M* | -0.0003 | -0.0003 | 0.0001 | -0.0011 | -0.0003 | 0.0000 | Condition | *F*_(2, 42)_ = 0.57, *p =* 0.77, ƞ^2^ = 0.03 |
|  |  |  | *SD* | 0.0010 | 0.0013 | 0.0008 | 0.0011 | 0.0005 | 0.0013 | Block | *F*_(1, 21)_ = 0.12, *p =* 0.73, ƞ^2^ = 0.01 |
|  | Girl | 12 | *M* | -0.0001 | 0.0001 | 0.0000 | 0.0000 | 0.0000 | 0.0003 | Gender | *F*_(1, 21)_ = 3.21, *p =* 0.09, ƞ^2^ = 0.13 |
|  |  |  | *SD* | 0.0007 | 0.0007 | 0.0012 | 0.0009 | 0.0012 | 0.0014 | Condition*Block | *F*_(2, 42)_ = 3.78, *p =* 0.03, ƞ^2^ = 0.15 |
|  | Total | 23 | *M* | -0.0002 | -0.0001 | 0.0000 | -0.0006 | -0.0001 | 0.0001 | Condition*Gender | *F*_(2, 42)_ = 0.16, *p =* 0.86, ƞ^2^ = 0.01 |
|  |  |  | *SD* | 0.0009 | 0.0010 | 0.0010 | 0.0012 | 0.0009 | 0.0014 | Block*Gender | *F*_(1, 21)_ = 1.59, *p =* 0.22, ƞ^2^ = 0.07 |
|  |  |  |  |  |  |  |  |  |  | Condition*Block*Gender | *F*_(2, 42)_ = 1.76, *p =* 0.19, ƞ^2^ = 0.08 |
| 9 | Boy | 12 | *M* | 0.0001 | -0.0001 | -0.0004 | -0.0013 | 0.0004 | 0.0001 | Condition | *F*_(2, 44)_ = 2.85, *p =* 0.07, ƞ^2^ = 0.012 |
|  |  |  | *SD* | 0.0006 | 0.0011 | 0.0020 | 0.0016 | 0.0013 | 0.0019 | Block | *F*_(1, 22)_ = 5.32, *p =* 0.03, ƞ^2^ = 0.20 |
|  | Girl | 12 | *M* | 0.0002 | 0.0003 | 0.0001 | 0.0000 | 0.0004 | 0.0001 | Gender | *F*_(1, 22)_ = 3.56, *p =* 0.07, ƞ^2^ = 0.14 |
|  |  |  | *SD* | 0.0005 | 0.0012 | 0.0008 | 0.0008 | 0.0004 | 0.0013 | Condition*Block | *F*_(2, 44)_ = 0.42, *p =* 0.66, ƞ^2^ = 0.02 |
|  | Total | 24 | *M* | 0.0002 | 0.0001 | -0.0002 | -0.0006 | 0.0004 | 0.0001 | Condition*Gender | *F*_(2, 44)_ = 0.98, *p =* 0.38, ƞ^2^ = 0.04 |
|  |  |  | *SD* | 0.0006 | 0.0011 | 0.0015 | 0.0014 | 0.0009 | 0.0016 | Block*Gender | *F*_(1, 22)_ = 1.80, *p =* 0.19, ƞ^2^ = 0.08 |
|  |  |  |  |  |  |  |  |  |  | Condition*Block*Gender | *F*_(2, 44)_ = 0.56, *p =* 0.57, ƞ^2^ = 0.03 |
| 10 | Boy | 12 | *M* | -0.0002 | -0.0004 | 0.0005 | -0.0012 | 0.0002 | -0.0004 | Condition | *F*_(2, 44)_ = 1.58, *p =* 0.22, ƞ^2^ = 0.07 |
|  |  |  | *SD* | 0.0008 | 0.0008 | 0.0006 | 0.0011 | 0.0008 | 0.0013 | Block | *F*_(1, 22)_ = 10.72, *p =* 0.003, ƞ^2^ = 0.33 |
|  | Girl | 12 | *M* | 0.0002 | 0.0002 | 0.0001 | -0.0005 | 0.0003 | 0.0000 | Gender | *F*_(1, 22)_ = 3.97, *p =* 0.06, ƞ^2^ = 0.15 |
|  |  |  | *SD* | 0.0006 | 0.0007 | 0.0008 | 0.0014 | 0.0006 | 0.0012 | Condition*Block | *F*_(2, 44)_ = 3.77, *p =* 0.03, ƞ^2^ = 0.15 |
|  | Total | 24 | *M* | 0.0000 | -0.0001 | 0.0003 | -0.0008 | 0.0003 | -0.0002 | Condition*Gender | *F*_(2, 44)_ = 0.34, *p =* 0.72, ƞ^2^ = 0.02 |
|  |  |  | *SD* | 0.0007 | 0.0008 | 0.0007 | 0.0013 | 0.0007 | 0.0012 | Block*Gender | *F*_(1, 22)_ = 2.77, *p =* 0.11, ƞ^2^ = 0.11 |
|  |  |  |  |  |  |  |  |  |  | Condition*Block*Gender | *F*_(2, 44)_ = 0.74, *p =* 0.48, ƞ^2^ = 0.03 |

*Note.* Ch = Channel; M = mean; SD = standard deviation
